# Added Sugar Sources and Their Impact on Diet Quality and Nutrient Intakes: An Analysis Across Added Sugar Intake Levels in United States Children and Adults

**DOI:** 10.1016/j.cdnut.2025.107566

**Published:** 2025-10-03

**Authors:** Michelle Tucker, Bibiana Garcia-Jackson, Victor L Fulgoni

**Affiliations:** 1 Bell Institute of Health and Nutrition, General Mills, Inc, Minneapolis, MN, United States; 2 Nutrition Impact, LLC, Battle Creek, MI, United States

**Keywords:** added sugars, Healthy Eating Index, nutrients of public health concern, shortfall nutrients, calcium, fiber, potassium, vitamin D, ready-to-eat cereals, NHANES

## Abstract

**Background:**

Added sugars (AS) are considered free sugars that are added to foods, and the Dietary Guidelines for Americans 2020‒2025 recommend limiting their intake to <10% calories.

**Objectives:**

To evaluate diet quality and nutrient adequacy at increasing AS intake levels.

**Methods:**

Two-day 24-h dietary recall data from the National Health and Nutrition Examination Survey 2011–2018 were used for intake assessment, and AS intake categories were defined as <10%, 10–15% and >15% of calories from AS.

**Results:**

About one-third of the children were in each AS intake category, although nearly half of the adult population was in the <10% calories from AS category, and the mean AS intake was 13.4% calories in children and 12.2% calories in adults. With increasing AS calories, diet quality (Healthy Eating Index 2020 scores), and intakes and adequacies for nutrients of public health concern (calcium, vitamin D, fiber, and potassium), and numerous other nutrients decreased among both children and adults. “Soft drinks,” “fruit drinks,” “tea,” “cookies and brownies,” “cakes and pies,” “ice creams and frozen dairy desserts,” and “candies” were consistently among the top 10 sources of AS for both children and adults. The top 10 sources of AS contributed ∼70% of AS in the diets of children and adults, but only 8‒11% and 2‒7% of daily intakes of nutrients of public health concern, respectively. Ready-to-eat cereals were the fourth and the ninth sources of AS and provided 6.1% and 3.1% AS, and 2.7‒9.9% and 1.5‒6.4% nutrients of public health concern in the diet. With increasing AS calories, the contribution of “soft drinks,” “fruit drinks,” and “tea” to AS increased, whereas the contribution of ready-to-eat cereals to AS decreased in both children and adults, respectively.

**Conclusions:**

Higher AS intakes were associated with lower diet quality, lower nutrient intakes, and lower nutrient adequacy in both children and adults; however, recommendations to reduce AS should focus on reducing AS from non-nutrient-dense sources, while recognizing the nutrient contributions of nutrient-dense foods and beverages containing AS. These findings can provide useful insights for policy and regulatory development related to nutrition and public health.

## Introduction

Added sugars (AS) are defined as the “sugars that are either added during the processing of foods, or packaged as such and include sugars (mono- and disaccharides), sugars from syrups and honey, and sugars from concentrated fruit/vegetable juices” by the 2020 Dietary Guidelines Advisory Committee [1]. Policy makers from around the world have proposed recommendations on AS. Dietary Guidelines for Americans 2020‒2025 (DGA) recommend limiting AS to <10% of total calories in healthy dietary patterns [2]. The WHO has recommended reducing intake of “free sugars” (inclusive of both AS and sugars naturally present in fruit juice) to <10% daily calories as a strong recommendation and further reduction to <5% daily calories as a conditional recommendation [3]. Quantitative recommendations and the establishment of the daily value for AS, as well as inclusion on the nutrition facts panel in the United States, have created a focus on AS sources without the context of broader nutrient contributions to the dietary pattern.

Current (2017‒2020 prepandemic) intakes of AS among United States children and adults are estimated to be ∼16.6 tsp eq/d (69.7 g/d ) [4]. Another recent analysis on NHANES 2011‒2018 also estimated AS intake among the United States population as 12.7% daily calories (68 g/d), with the highest (14.3% daily calories) being among adolescents/teens age 9‒18 y and the lowest (11.3% daily calories) being among older adults age 71+ y [5]. NHANES data indicate that ∼35% of children and 47% of adults consume <10% of calories from AS (AS10), which aligns with DGA recommendations [6,7].

Although these estimates of AS intake in the overall population are higher than the DGA recommendations, the data suggest that there has been a steady decline in AS intake among United States children age 2‒18 y from 17.3% daily calories (88.7 g/d) in 2001‒2002 to 13.6% daily calories (65.5 g/d) in 2017‒2018 [8]. Additionally, in adults age 19+ y, AS intakes have steadily declined during the last 2 decades from 14.8% daily calories (83.4 g/d) in 2001‒2002 to 12.4% daily calories (68.5 g/d) in 2017‒2018 [9].

Limited information exists on the association of AS with diet quality and micronutrient adequacy. We previously reported that increasing AS intake was associated with increasing prevalence of inadequacy for magnesium and vitamins C, D, and E among United States adults [10]. A range of AS intake exists in the United States population, with over one-third of children and nearly half of adults meeting DGA recommendations for AS (AS10), whereas approximately one-third exceed recommendations by 50% [>15% of calories from AS, (AS15)]. The remainder of the population falls between 10% and 15% of calories from AS (AS10‒15). Next to no information is available on the nutrient intakes, nutrient adequacy, and diet quality across these observed ranges of AS intakes. Furthermore, no studies have comprehensively evaluated food and beverage contributors to AS intakes and their nutritional contributions across this continuum. Such information is critical toward developing more effective, targeted public health nutrition strategies to reduce AS intake in the population subgroups that may benefit the most, without adversely impacting the intake of positive nutrients and food groups that help meet dietary recommendations.

The objective of the present study was to characterize diet quality and nutrient adequacy among children and adults with various concentrations of AS intake in the diet. Building upon previous studies that looked at characteristics of high-AS consumers [11] and micronutrient intakes of consumers with increasing AS intakes [10], we sought to examine diet quality across different groups of AS intakes and how top sources of AS were associated with changes in nutrient intakes and diet quality. We hypothesized that consumers with higher AS intakes would have lower diet quality and lower intakes of nutrient-dense sources of AS, such as ready-to-eat cereal (RTEC) and flavored dairy.

## Methods

### Database and participants

The NHANES, administered by the National Center for Health Statistics of the Centers for Disease Control and Prevention, is a robust program that aims to assess the overall nutrition and health status of the American population [12]. The survey, aiming to deliver population-level insights, employs a sophisticated multi-stage, probability-based sampling design to ensure a nationally representative sample and collects data annually and releases data in 2-y cycles. The data from 4 cycles of NHANES (2011–2012, 2013‒2014, 2015‒2016, and 2017‒2018) were combined for the present analysis. The combined sample included 26,526 participants age 2+ y, excluding pregnant/lactating females (*n* = 319) and those with unreliable or missing first or second day 24-h recall data (*n* = 9722) or with 0 calorie intake on either day of dietary recall (*n* = 21). The research ethics review board of the National Center for Health Statistics approved the survey protocol, and all participants or proxies provided written informed consent. NHANES has stringent consent protocols and procedures to ensure confidentiality and protection from identification. The present study was a secondary data analysis, which lacked personal identifiers; therefore, it was exempt from additional institutional review board approval.

### Dietary intakes

Dietary intake data were obtained from reliable 24-h dietary-recall interviews (2 d) using the USDA automated multiple-pass method and included a description and the amount of the individual foods and beverages consumed during the 24-h period before the interview (midnight to midnight) for each participant. Complete descriptions of the dietary-interview methods for NHANES are provided elsewhere [13].

Intake of AS was determined using the NHANES cycle-specific USDA Food Patterns Equivalent Database (FPED) [14]. AS were defined as “sugars that are added to foods as an ingredient during preparation, processing, or at the table” by FPED and did not include naturally occurring sugars, such as lactose present in milk and fructose present in whole or cut fruit, or 100% fruit juice. However, more recently, fruit juice concentrates used as ingredients were also assigned to AS by FPED. Intake of AS was evaluated as a percentage of total calories from AS for each day of dietary recall. Subjects were then placed into categories defined as <10% (AS10), 10-15% (AS10-15) and >15% (AS15) of calories from added sugars using the average of the two days of recall, as previously reported [15].

Nutrient intakes (only from foods and beverages, excluding dietary supplements) and food group intakes were determined using the NHANES cycle-specific USDA Food and Nutrient Database for Dietary Studies and FPED, respectively [14,16]. Of particular interest were intakes of *1*) “nutrients of public health concern” calcium, vitamin D, fiber, and potassium; *2*) “shortfall nutrients” magnesium, vitamins A, D, E, and C, and folate; and *3*) iron in adolescent girls and premenopausal females [2].

Diet quality scores were estimated using the USDA Healthy Eating Index (HEI) 2020 [17,18], which is composed of 13 components that reflect different food groups and key recommendations in the DGA [2]. The HEI 2020 scores were estimated using the mean of 2 d of dietary intake data. For analysis of sources of AS and nutrients from these sources, intakes from all food sources were summed for all the USDA 168 food categories in What We Eat In America (WWEIA) 2017‒2018, then these sums were multiplied by day 1 sample weights to obtain a population-weighted total intake for each food category. We then divided each individual food category intake by the total intake of energy, AS, or nutrients to provide a percentage contribution for each food category.

### Statistics

All analyses were performed using Statistical Analysis System 9.4 (SAS Institute) software, and the data were adjusted for the complex sampling design of NHANES, using appropriate survey weights, strata, and primary sampling units. Separate analyses were conducted for gender combined for children aged 2‒18 y (*n* = 9292) and adults aged 19+ y (*n* = 17,234). Additional analyses were also conducted for those 2‒4 y, 5‒8 y, 9‒13 y, 14‒18 y, 19‒30 y, 31‒59 y, and 60+ y. Demographic variables of interest included age, gender, self-defined race/ethnicity, poverty income ratio (ratio of household income to United States poverty level, using 3 groups: <1.35, 1.35‒1.85, and >1.85 % of the poverty level), physical activity (using 3 groups: sedentary, moderate and vigorous, based on responses to the physical activity questionnaire), and current smoking status.

To assess differences in demographics across the AS intake categories for those 2‒18 y and 19+ y, regression analyses were conducted to estimate a β coefficient for changes in demographic variables as AS intake as a percentage of calories increased. For all age groups, regression analyses were used to assess a linear trend in food group intakes, HEI total and subcomponent scores, and nutrient intakes across AS categories. All these analyses were adjusted for the complex sampling plan of NHANES using primary sample units, strata, and 2-d sampling weights.

The distributions of usual nutrient intakes from foods alone (intakes from dietary supplements not included) were estimated using the National Cancer Institute method (version 2.1) [19]. Given nutrients are consumed on most days by most individuals [20], the 1-part model was used for the usual intake estimates, including 2 d of intakes and 1-d sampling weights to obtain percentiles of intake and necessary variance estimates. Covariates used in the usual intake estimates were the day of the week of the 24-h dietary recall [coded as weekend (Friday to Sunday) or weekday (Monday to Thursday)] and the sequence of recall (first or second). The percentage of the population below the estimated average requirement (EAR) or above the adequate intake (AI) was determined using the cut-point method, except for iron, for which the probability method was used [21]. Z-tests were used to evaluate differences in AS groups. *P* < 0.05 was deemed significant for all analyses.

## Results

### Demographics

Table 1 provides the results of the regression analyses of AS intakes on demographic variables. As the percentage of calories from AS increased (from AS10 to AS15), there was a significant decrease in percentage Hispanics and percentage non-Hispanic Asians and a increase in % current smokers among children (2‒18 y) and adults (19+ y). Additionally, there was a significant increase in age for children, an increase in % non-Hispanic Black and a decrease in vigorous physical activity and percentage in the poverty income ratio >1.85 group in adults, with an increasing percentage of calories from AS. There were no other significant changes in demographic characteristics with increasing percentage of calories from AS.

**TABLE 1:** Regression of added sugar intakes as a percentage of calories on demographic variables using 2-d mean intakes: NHANES 2011‒2018 data

	Children (aged 2‒18 y)		Adults (aged 19+ y)	
	β ± SE	*P* value	β ± SE	*P* value
Age, years	0.63 ± 0.09	<0.0001	‒0.03 ± 0.04	0.3877
Age squared	‒0.02 ± 0.005	<0.0001	‒0.0001 ± 0.0004	0.8602
Gender (% male)	0.28 ± 0.23	0.2344	‒0.21 ± 0.20	0.3045
Ethnicity				
Non-Hispanic White (%)^1^	0		0	
Hispanic (%)	‒2.04 ± 0.33	<0.0001	‒1.06 ± 0.27	0.0003
Non-Hispanic Black (%)	‒0.06 ± 0.34	0.8596	0.96 ± 0.29	0.0017
Non-Hispanic Asian (%)	‒4.19 ± 0.38	<0.0001	‒4.04 ± 0.26	<0.0001
Other (%)	‒1.12 ± 0.53	0.0390	0.38 ± 0.45	0.4045
Poverty income ratio				
<1.35 (%)^1^	0		0	
1.35 ≤ 1.85 (%)	0.41 ± 0.36	0.2574	‒0.29 ± 0.31	0.3417
>1.85 (%)	‒0.18 ± 0.27	0.5055	‒1.68 ± 0.29	<0.0001
Physical activity				
Sedentary (%)^1^	0		0	
Moderate (%)	0.20 ± 0.41	0.6337	‒0.42 ± 0.30	0.1684
Vigorous (%)	‒0.29 ± 0.37	0.4318	‒1.25 ± 0.25	<0.0001
Current smoking status (%)				
No^1^	0		0	
Yes	10.1 ± 2.8	0.0007	3.44 ± 0.30	<0.0001
BMI (kg/m^2^)	0.0003 ± 0.0268	0.9908	0.02 ± 0.02	0.1102

Regression analysis was conducted with AS percentage of calories as a continuous variable determined as the mean of 2 d of intake adjusting for the complex sample design of NHANES and using 2-d dietary weights.

Abbreviations: BMI, body mass index; NHANES, National Health and Nutrition Examination Survey; SE, standard error.

1 Reference group.

The 2-d mean intake of AS was 13.4 ± 0.1% and 12.2 ± 0.1% calories, and the median intake of AS was 12.5 ± 0.1% and 10.5 ± 0.1% calories among all children and all adults, respectively. In all children, about one-third of the population was in each of the AS intake categories (AS10, AS10‒15, and AS15). However, nearly half of the adult population (47%) was in AS10, whereas slightly less than a fourth (23.5%) was in AS10‒15 and slightly over a fourth (29.5%) was in AS15.

The intakes of whole grain, total dairy, total fruit, total vegetables, and total protein foods decreased with increasing calories from AS categories for both all children and all adults. The differences between the AS10 and AS15 categories were ‒28% and ‒39% for whole grain, ‒18% and ‒5% for total dairy, ‒40% and ‒39% for total fruit, ‒18% and ‒29% for total vegetables, and ‒14% and ‒20% for total protein foods among all children and all adults, respectively. Added sugar intakes were significantly higher in the AS15 subgroup compared to the other 2 subgroups, and the differences between AS10 and AS15 were +217% and +298% for children and adults, respectively (Table 2). Similar results were found for children 2‒4 y, 5‒8 y, 9‒13 y, and 14‒18 y (Supplemental Table 1) and whereas in general similar results were seen in adults 19‒30 y, 31‒59 y, and 60+ y, total dairy in those 31‒59 and 60+ y and total grains for those 60+ y were not different across AS categories (Supplemental Table 1).

**TABLE 2:** Intakes (2 d mean) for food groups by percentage of calories from added sugars (<10%, 10‒15%, and >15%) in children and adults - NHANES 2011‒2018 data, gender combined population

	Calories from AS (AS intake categories)			*P*-_linear trend_
	<10% (AS10)	10‒15% (AS10‒15)	>15% (AS15)	
**Children (aged 2‒18 y)**				
AS (tsp eq)	7.60 ± 0.11	14.5 ± 0.2	24.1 ± 0.4	<0.0001
Total dairy (cup eq)	2.18 ± 0.05	2.06 ± 0.03	1.78 ± 0.04	<0.0001
Total fruits (cup eq)	1.36 ± 0.04	1.15 ± 0.03	0.81 ± 0.03	<0.0001
Total grains (oz eq)	6.66 ± 0.09	6.88 ± 0.10	6.21 ± 0.08	<0.0001
Whole grains (oz eq)	0.97 ± 0.03	0.92 ± 0.02	0.70 ± 0.03	<0.0001
Total protein foods (oz eq)	4.41 ± 0.11	4.17 ± 0.09	3.80 ± 0.07	<0.0001
Total vegetables^1^ (cup eq)	0.97 ± 0.02	0.87 ± 0.02	0.80 ± 0.02	<0.0001
**Adults (aged 19+ y)**				
AS (tsp eq)	7.57 ± 0.08	16.5 ± 0.1	30.1 ± 0.4	<0.0001
Total dairy (cup eq)	1.53 ± 0.02	1.63 ± 0.04	1.46 ± 0.03	0.0004
Total fruits (cup eq)	1.12 ± 0.02	0.96 ± 0.03	0.68 ± 0.03	<0.0001
Total grains (oz eq)	6.44 ± 0.07	6.77 ± 0.09	6.21 ± 0.09	0.0004
Whole grains (oz eq)	1.09 ± 0.03	1.00 ± 0.03	0.66 ± 0.03	<0.0001
Total protein foods (oz eq)	6.73 ± 0.07	6.18 ± 0.06	5.40 ± 0.07	<0.0001
Total vegetables^1^ (cup eq)	1.75 ± 0.02	1.52 ± 0.02	1.25 ± 0.02	<0.0001

Regression analysis was conducted with AS a percentage of calories as a continuous variable determined as the mean of 2 d of intake, adjusting for the complex sample design of NHANES and using 2-d dietary weights.

Abbreviation: AS, added sugar; AS10, <10% calories from added sugar; AS10‒15, 10‒15% calories from added sugar; AS15, >15% calories from added sugar; NHANES, National Health and Nutrition Examination Survey.

1 Total vegetables exclude legumes.

Diet quality, assessed as HEI 2020 scores, decreased with increasing calories from AS among all children and all adults. The difference in the HEI 2020 total scores between the AS10 and AS15 categories for all children and all adults was ‒17% and ‒21%, respectively. With increasing calories from AS among all children and all adults, the HEI 2020 subcomponent scores for total vegetables, greens and beans, total fruit, whole fruit, whole grains, dairy, total protein foods, seafood, and plant protein gradually decreased, and subcomponent scores for sodium, refined grain, and saturated fat gradually increased. The subcomponent scores for AS dropped 6.2 points in all children and 6.9 points for all adults between AS10 and AS15. The HEI 2020 subcomponent score for the fatty acid ratio also decreased with increasing intake of AS calories in all adults, whereas it did not change in all children (Table 3). Similar results in changes in HEI total and subcomponent scores across AS categories were found for all other age groups with the difference in total HEI scores between AS10 and AS15 for those 2‒4 y, 5‒8 y, 9‒13 y,14‒18 y, 19‒30 y, 31‒59 y, and 60+ y being ‒12%, ‒16%, ‒16%, ‒16%, ‒21%, ‒20%, ‒17%, respectively (Supplemental Table 2).

**TABLE 3:** Healthy eating index (2 d mean) by percentage of calories from added sugars in children and adults - NHANES 2011‒2018 data, gender combined population

	Calories from AS (AS intake categories)			*P*-_linear trend_
	<10% (AS10)	10‒15% (AS10‒15)	>15% (AS15)	
**Children (aged 2‒18 y)**				
HEI 2020 total score	55.4 ± 0.4	51.1 ± 0.3	46.1 ± 0.4	<0.0001
Component score for total fruits	3.46 ± 0.06	3.02 ± 0.06	2.27 ± 0.08	<0.0001
Component score for whole fruits	3.39 ± 0.07	2.96 ± 0.06	2.26 ± 0.08	<0.0001
Component score for total vegetables	2.61 ± 0.04	2.22 ± 0.04	2.01 ± 0.03	<0.0001
Component score for greens and beans	1.64 ± 0.06	1.21 ± 0.06	1.00 ± 0.05	<0.0001
Component score for whole grains	3.60 ± 0.11	3.22 ± 0.08	2.49 ± 0.08	<0.0001
Component score for dairy	7.76 ± 0.10	7.40 ± 0.09	6.59 ± 0.11	<0.0001
Component score for total protein foods	4.17 ± 0.03	3.93 ± 0.05	3.67 ± 0.04	<0.0001
Component score for seafood and plant protein	2.43 ± 0.06	2.12 ± 0.06	1.79 ± 0.05	<0.0001
Component score for fatty acid ratio	3.78 ± 0.13	3.62 ± 0.09	3.75 ± 0.09	0.9963
Component score for refined grains	4.57 ± 0.10	4.71 ± 0.11	5.58 ± 0.10	<0.0001
Component score for sodium	3.73 ± 0.08	4.61 ± 0.09	5.59 ± 0.10	<0.0001
Component score for saturated fat	4.84 ± 0.14	5.05 ± 0.09	5.89 ± 0.08	<0.0001
Component score for AS	9.39 ± 0.02	6.98 ± 0.03	3.21 ± 0.04	<0.0001
**Adults (aged 19+ y)**				
HEI 2020 total score	58.3 ± 0.3	53.7 ± 0.4	46.7 ± 0.3	<0.0001
Component score for total fruits	2.65 ± 0.04	2.35 ± 0.06	1.71 ± 0.04	<0.0001
Component score for whole fruits	2.89 ± 0.05	2.55 ± 0.07	1.84 ± 0.05	<0.0001
Component score for total vegetables	3.66 ± 0.02	3.21 ± 0.03	2.71 ± 0.04	<0.0001
Component score for greens and beans	2.44 ± 0.04	2.02 ± 0.06	1.48 ± 0.05	<0.0001
Component score for whole grains	3.40 ± 0.07	3.01 ± 0.09	2.03 ± 0.06	<0.0001
Component score for dairy	5.41 ± 0.05	5.45 ± 0.08	4.93 ± 0.07	<0.0001
Component score for total protein foods	4.67 ± 0.01	4.53 ± 0.02	4.23 ± 0.02	<0.0001
Component score for seafood and plant protein	3.29 ± 0.04	3.00 ± 0.05	2.38 ± 0.05	<0.0001
Component score for fatty acid ratio	5.39 ± 0.07	4.84 ± 0.08	4.42 ± 0.06	<0.0001
Component score for refined grains	6.08 ± 0.08	6.21 ± 0.09	6.67 ± 0.07	<0.0001
Component score for sodium	3.18 ± 0.06	4.07 ± 0.09	5.35 ± 0.08	<0.0001
Component score for saturated fat	5.68 ± 0.08	5.47 ± 0.07	6.20 ± 0.07	<0.0001
Component score for AS	9.58 ± 0.01	7.02 ± 0.02	2.71 ± 0.04	<0.0001

Regression analysis was conducted with AS as a percentage of calories as a continuous variable determined as the mean of 2 d of intake, adjusting for the complex sample design of NHANES and using 2-d dietary weights.

Abbreviations: AS, added sugar; AS10, <10% calories from added sugar; AS10‒15, 10‒15% calories from added sugar; AS15, >15% calories from added sugar; HEI, Healthy Eating Index; NHANES, National Health and Nutrition Examination Survey.

Intakes of nutrients of public health concern (calcium, vitamin D, fiber, and potassium) also decreased with increasing calories from AS among all children and all adults. The differences between the AS10 and AS15 categories were ‒15% and ‒9% for calcium, ‒21% and ‒15% for vitamin D, ‒19% and ‒24% for dietary fiber, and ‒12% and ‒16% for potassium among children and adults, respectively (Table 4). The percentage of all children and all adults with intakes below the EAR for nutrients of public health concern also increased with increasing calories from AS (Table 5). Except for niacin, vitamin B_6_, vitamin B_12_, and vitamin E in all children and vitamin B_12_ in all adults, intake of all of the other nutrients evaluated decreased with increasing calories from AS among all children and all adults (Table 4). Other than selenium, riboflavin, niacin, vitamin B_12_, and sodium in all children and vitamin B_12_ in all adults, the percentage of the population below the EAR increased or above the AI decreased for all other nutrients evaluated, comparing those in AS10 to those in AS15 (Table 5). In general, similar changes in nutrient intake with increasing calories from AS were seen in those 5‒8 y, 9‒13 y, 14‒18 y, 19‒30 y, and 31‒59 y. In those 2‒4 y intakes of copper, iron, riboflavin, niacin, folate, vitamin B_12_, vitamin C, vitamin E, and sodium were unchanged with increasing calories from AS. In addition, intakes of iron, thiamin, niacin, vitamin B_6_, and vitamin B_12_ were unchanged with increasing calories from AS in those 60+ y (Supplemental Table 3).

**TABLE 4:** Nutrient intakes (2 d mean) by a percentage of calories from added sugars in children and adults – from NHANES 2011‒2018

	Calories from AS (AS intake categories)			*P*-_linear trend_
	<10% (AS10)	10‒15% (AS10‒15)	>15% (AS15)	
**Children (aged 2‒18 y)**				
Calcium (mg)	1064 ± 18	1027 ± 12	907 ± 16	<0.0001
Copper (mg)	0.95 ± 0.02	0.94 ± 0.02	0.86 ± 0.01	<0.0001
Iron (mg)	13.7 ± 0.2	14.4 ± 0.2	13.3 ± 0.2	0.0430
Magnesium (mg)	245 ± 4	234 ± 2	214 ± 2	<0.0001
Phosphorus (mg)	1307 ± 18	1272 ± 11	1149 ± 14	<0.0001
Selenium (*μ*g)	101 ± 1	97.2 ± 1.2	85.7 ± 1.0	<0.0001
Zinc (mg)	10.1 ± 0.2	9.99 ± 0.12	9.28 ± 0.15	0.0001
Vitamin A, RE (*μ*g)	628 ± 13	620 ± 14	543 ± 11	<0.0001
Thiamin (mg)	1.55 ± 0.02	1.58 ± 0.02	1.46 ± 0.02	0.0002
Riboflavin (mg)	1.95 ± 0.04	1.96 ± 0.02	1.82 ± 0.03	0.0037
Niacin (mg)	21.4 ± 0.4	21.8 ± 0.3	21.1 ± 0.4	0.9186
Folate, DFE (*μ*g)	513 ± 9	532 ± 10	482 ± 10	0.0011
Vitamin B_6_ (mg)	1.78 ± 0.04	1.77 ± 0.03	1.70 ± 0.04	0.5750
Vitamin B_12_ (*μ*g)	4.74 ± 0.12	4.81 ± 0.12	4.64 ± 0.13	0.8051
Vitamin C (mg)	78.5 ± 2.2	73.5 ± 2.1	67.7 ± 1.7	0.0001
Vitamin D (*μ*g)	5.93 ± 0.14	5.43 ± 0.10	4.71 ± 0.12	<0.0001
Vitamin E, ATE (mg)	7.18 ± 0.28	7.24 ± 0.12	6.78 ± 0.11	0.0682
Dietary fiber (g)	15.4 ± 0.3	14.5 ± 0.2	12.5 ± 0.1	<0.0001
Potassium (mg)	2263 ± 28	2155 ± 19	1983 ± 24	<0.0001
Sodium (mg)	2981 ± 35	3002 ± 32	2841 ± 37	0.0192
Choline (mg)	269 ± 4	251 ± 3	227 ± 3	<0.0001
**Adults (aged 19+ y)**				
Calcium (mg)	979 ± 9	1004 ± 15	887 ± 13	<0.0001
Copper (mg)	1.34 ± 0.02	1.26 ± 0.02	1.07 ± 0.01	<0.0001
Iron (mg)	14.6 ± 0.1	15.2 ± 0.2	14.0 ± 0.2	<0.0001
Magnesium (mg)	328 ± 3	307 ± 3	258 ± 3	<0.0001
Phosphorus (mg)	1431 ± 11	1410 ± 15	1267 ± 14	<0.0001
Selenium (*μ*g)	122 ± 1	115 ± 1	103 ± 1	<0.0001
Zinc (mg)	11.5 ± 0.1	11.5 ± 0.1	10.3 ± 0.1	<0.0001
Vitamin A, RE (*μ*g)	688 ± 13	670 ± 15	562 ± 11	<0.0001
Thiamin (mg)	1.63 ± 0.01	1.67 ± 0.02	1.51 ± 0.02	<0.0001
Riboflavin (mg)	2.15 ± 0.03	2.22 ± 0.03	2.03 ± 0.03	0.0001
Niacin (mg)	26.3 ± 0.3	26.5 ± 0.3	25.0 ± 0.4	0.0006
Folate, DFE (*μ*g)	538 ± 6	545 ± 8	488 ± 9	<0.0001
Vitamin B_6_ (mg)	2.19 ± 0.03	2.21 ± 0.04	2.05 ± 0.04	0.0023
Vitamin B_12_ (*μ*g)	4.90 ± 0.09	5.21 ± 0.11	5.00 ± 0.09	0.4896
Vitamin C (mg)	86.4 ± 1.4	81.4 ± 1.7	66.3 ± 1.6	<0.0001
Vitamin D (*μ*g)	4.78 ± 0.08	4.82 ± 0.11	4.05 ± 0.09	<0.0001
Vitamin E, ATE (mg)	9.53 ± 0.14	9.39 ± 0.16	7.98 ± 0.13	<0.0001
Dietary fiber (g)	18.9 ± 0.2	17.5 ± 0.2	14.3 ± 0.2	<0.0001
Potassium (mg)	2795 ± 24	2699 ± 26	2360 ± 24	<0.0001
Sodium (mg)	3530 ± 26	3550 ± 34	3293 ± 38	<0.0001
Choline (mg)	356 ± 3	334 ± 3	292 ± 3	<0.0001

Regression analysis was conducted with AS as a percentage of calories as a continuous variable determined as the mean of 2 d of intake, adjusting for the complex sample design of NHANES and using 2-d dietary weights.

Abbreviations: AS, added sugar; AS10, <10% calories from added sugar; AS10‒15, 10‒15% calories from added sugar; AS15, >15% calories from added sugar; ATE, α-tocopherol equivalents; DFE, dietary folate equivalents; NHANES, National Health and Nutrition Examination Survey; RE, retinol activity equivalents.

**TABLE 5:** Percentage of the population below the Estimated Average Requirement/above the Adequate Intake for those with <10%, 10‒15%, and >15% of calories as added sugars in children and adults - NHANES 2011‒2018 data, gender combined population

	Calories from AS (AS intake categories)			*P*-values for comparison		
	<10% (AS10)	10‒15% (AS10‒15)	>15% (AS15)	<10% vs. 10‒15% (AS10 vs. AS10‒15)	<10% vs. >15% (AS10 vs. AS15)	10‒15% vs. >15% (AS10‒15 vs. AS15)
**Children (aged 2‒18 y)**						
% below EAR						
Calcium	36.4 ± 1.5	42.9 ± 1.9	60.7 ± 2.1	0.0064	<0.0001	<0.0001
Copper	2.91 ± 0.60	3.08 ± 0.55	7.25 ± 1.18	0.8349	0.0010	0.0014
Iron	2.05 ± 0.32	2.19 ± 0.37	3.99 ± 0.72	0.7720	0.0142	0.0268
Magnesium	26.8 ± 1.5	31.9 ± 1.3	44.7 ± 1.6	0.0111	<0.0001	<0.0001
Phosphorus	10.4 ± 1.2	10.5 ± 1.2	23.7 ± 1.9	0.9653	<0.0001	<0.0001
Selenium	0.02 ± 0.02	0.02 ± 0.02	0.02 ± 0.04	0.9393	0.8959	0.9295
Zinc	7.84 ± 1.24	7.00 ± 1.42	17.2 ± 2.0	0.6551	0.0001	<0.0001
Vitamin A, RE	20.1 ± 1.9	19.3 ± 1.7	34.3 ± 1.8	0.7520	<0.0001	<0.0001
Thiamin	0.73 ± 0.21	0.63 ± 0.29	3.24 ± 0.87	0.7648	0.0050	0.0043
Riboflavin	1.01 ± 0.27	0.41 ± 0.21	2.08 ± 0.57	0.0788	0.0903	0.0061
Niacin	0.17 ± 0.09	0.06 ± 0.05	0.79 ± 0.37	0.2851	0.1051	0.0518
Folate, DFE	2.15 ± 0.54	2.75 ± 0.78	7.34 ± 1.50	0.5257	0.0011	0.0065
Vitamin B_6_	1.42 ± 0.42	0.69 ± 0.34	5.16 ± 1.17	0.1786	0.0027	0.0003
Vitamin B_12_	1.60 ± 0.46	1.02 ± 0.40	1.95 ± 0.63	0.3422	0.6640	0.2191
Vitamin C	15.8 ± 1.7	18.7 ± 1.8	26.8 ± 2.5	0.2446	0.0003	0.0092
Vitamin D	89.2 ± 1.3	94.9 ± 0.9	96.7 ± 0.8	0.0005	<0.0001	0.1294
Vitamin E, ATE	62.0 ± 2.4	60.7 ± 2.0	72.3 ± 2.0	0.6797	0.0008	<0.0001
% above AI						
Dietary fiber	3.60 ± 0.84	0.95 ± 0.34	0.07 ± 0.05	0.0035	<0.0001	0.0095
Potassium	42.7 ± 2.2	32.1 ± 2.2	18.4 ± 1.9	0.0008	<0.0001	<0.0001
Sodium	99.9 ± 0.04	99.9 ± 0.04	99.8 ± 0.1	0.9496	0.2015	0.1885
Choline	31.0 ± 1.5	21.5 ± 1.7	10.5 ± 1.2	<0.0001	<0.0001	<0.0001
**Adults (aged 19+ y)**						
% below EAR						
Calcium	41.2 ± 0.9	38.1 ± 1.7	49.6 ± 1.5	0.1161	<0.0001	<0.0001
Copper	5.07 ± 0.52	3.54 ± 0.64	13.3 ± 1.1	0.0629	<0.0001	<0.0001
Iron	4.23 ± 0.29	4.01 ± 0.44	7.62 ± 0.60	0.6699	<0.0001	<0.0001
Magnesium	42.1 ± 1.1	50.6 ± 1.4	70.3 ± 1.2	<0.0001	<0.0001	<0.0001
Phosphorus	0.55 ± 0.12	0.17 ± 0.08	1.44 ± 0.40	0.0056	0.0327	0.0018
Selenium	0.34 ± 0.10	0.12 ± 0.08	1.25 ± 0.37	0.0785	0.0163	0.0025
Zinc	14.5 ± 1.0	12.9 ± 1.7	24.2 ± 1.3	0.4073	<0.0001	<0.0001
Vitamin A, RE	38.1 ± 1.4	40.9 ± 2.1	55.8 ± 1.6	0.2818	<0.0001	<0.0001
Thiamin	5.84 ± 0.57	4.18 ± 0.82	10.0 ± 1.1	0.0949	0.0007	<0.0001
Riboflavin	2.51 ± 0.34	1.38 ± 0.32	5.64 ± 0.73	0.0148	0.0001	<0.0001
Niacin	0.77 ± 0.18	0.66 ± 0.22	3.10 ± 0.66	0.7192	0.0007	0.0005
Folate, DFE	10.2 ± 0.9	10.2 ± 1.3	19.6 ± 1.5	0.9717	<0.0001	<0.0001
Vitamin B_6_	7.60 ± 0.68	9.20 ± 1.50	17.0 ± 1.4	0.3311	<0.0001	0.0002
Vitamin B_12_	4.81 ± 0.76	3.85 ± 0.92	6.97 ± 0.88	0.4227	0.0641	0.0146
Vitamin C	41.1 ± 1.4	45.9 ± 1.5	59.4 ± 1.5	0.0217	<0.0001	<0.0001
Vitamin D	94.5 ± 0.7	95.1 ± 0.7	96.9 ± 0.5	0.5529	0.0065	0.0370
Vitamin E, ATE	74.4 ± 1.2	79.6 ± 1.8	87.7 ± 1.3	0.0153	<0.0001	0.0002
% above AI						
Dietary fiber	13.7 ± 0.9	6.66 ± 1.0	1.76 ± 0.3	<0.0001	<0.0001	<0.0001
Potassium	39.1 ± 1.1	31.3 ± 1.5	19.0 ± 1.2	<0.0001	<0.0001	<0.0001
Sodium	99.4 ± 0.1	99.8 ± 0.1	98.6 ± 0.3	0.0125	0.0441	0.0011
Choline	13.1 ± 0.9	4.29 ± 1.02	3.18 ± 0.7	<0.0001	<0.0001	0.3611

Regression analysis was conducted assessing AS as categorical variables (AS10, AS10‒15 and AS>15) determined as the mean of 2 d of intake adjusting for the complex sample design of NHANES and using 2-d dietary weights with Z-tests to evaluate differences in groups.

Abbreviations: AI, adequate intake; AS, added sugar; AS, added sugar; AS10, <10% calories from added sugar; AS10‒15, 10‒15% calories from added sugar; AS15, >15% calories from added sugar; ATE, α tocopherol equivalent; DFE, dietary folate equivalent; EAR, estimated average requirement; NHANES, National Health and Nutrition Examination Survey; RE, retinol activity equivalents.

Except for selenium, riboflavin, niacin, vitamin B_12_, and sodium, the percentage of the population below the EAR increased or above the AI decreased in all children, comparing those in AS15 with those in AS10. In all adults, except for vitamin B_12_, the percentage of the population below the EAR increased, or above the AI decreased, comparing those in AS15 with those in AS10.

In those 2‒4 y and 5‒8 y the percentage of the population below the EAR increased or above the AI decreased for calcium, vitamin D, dietary fiber, potassium, and choline, comparing those in AS15 with those in AS10. Those 9‒13 y were similar to those 2‒8 y except that the percentage of the population below the EAR for phosphorus was also increased, comparing those in AS15 with those in AS10. Although the results in those 19‒30 and 31‒50 were generally similar to those for all adults, in those 60+ y the percentage of the population below the EAR for calcium, iron, vitamin B_12_, and vitamin D were not different comparing those in AS15 with those in AS10 (Supplemental Table 4).

All children consumed 16.8 tsp (70.6 g) of AS per day, with 109 out of the total 168 USDA food categories contributing to that daily amount. Meanwhile, all adults consumed 17.3 tsp (72.7 g) of AS daily, with 113 out of the 168 USDA food categories contributing to that amount. The top 10 sources of AS contributed ∼70%, or 11.6 tsp (48.7 g/d) of AS in the diets of all children and all adults (Table 6). “Soft drinks,” “fruit drinks,” “tea,” “cookies and brownies,” “cakes and pies,” “ice cream and frozen dairy desserts,” and “candies” were among the top 10 sources of AS for both children and adults.

**TABLE 6:** Top 10 sources of added sugars and their contribution to daily sugar intake by sugar intake categories

	Calories from AS (AS intake categories)			
	All	<10% (AS10)	10‒15% (AS10‒15)	>15% (AS15)
**Children (aged 2‒18 y)**				
Soft drinks	17.3	9.26	14.3	21.3
Fruit drinks	11.8	7.50	10.5	13.7
Cookies and brownies	7.22	9.16	7.72	6.36
High sugar RTEC	5.44	8.37	6.11	4.21
Candy not containing chocolate	5.33	3.87	4.87	6.02
Ice cream	5.11	5.70	5.10	4.93
Tea	4.73	2.51	4.00	5.80
Cakes and pies	4.62	NS (2.23)	4.65	5.30
Jams, syrups, toppings	3.72	3.60	4.00	3.59
Doughnuts, sweet rolls, pastries	3.43	4.40	4.03	NS (2.80)
Sport and energy drinks	NS (2.71)	NS (1.54)	NS (2.40)	3.23
Yeast bread	NS (1.01)	2.28	NS (1.15)	NS (0.56)
Total of top 10 sources	68.7	56.6	65.3	74.4
**Adults (aged 19+ y)**				
Soft drinks	24.5	9.29	17.6	33.8
Tea	8.06	3.90	5.16	11.0
Cakes and pies	5.91	5.47	7.05	5.59
Fruit drinks	5.62	3.51	5.72	6.45
Cookies and brownies	5.12	7.56	6.56	3.49
Sugars and honey	4.96	5.22	5.23	4.73
Ice cream	4.30	5.28	5.32	3.45
Candy containing chocolate	3.29	3.75	4.61	2.53
Sport and energy drinks	3.09	NS (1.74)	3.04	3.66
Candy not containing chocolate	2.61	NS (2.30)	NS (2.28)	2.87
Jams, syrups, toppings	NS (2.49)	NS (3.13)	3.00	NS (2.01)
High Sugar RTEC	NS (2.37)	3.70	NS (2.74)	NS (1.66)
Doughnuts, sweet rolls, pastries	NS (2.29)	3.15	NS (2.81)	NS (1.72)
Total top 10	67.5	50.8	63.3	77.6

Food sources were summed by subject and then multiplied day 1 sample weights to obtain a population-weighted total intake of AS; percentage contribution for each food category was calculated as the individual food category divided by the total intake.

High sugar RTEC, higher sugar (>21.2 g/100 g).Abbreviations: AS, added sugar; AS10, <10% calories from added sugar; AS10‒15, 10‒15% calories from added sugar; AS15, >15% calories from added sugar; NS, not in the top 10 sources of added sugars and not included in top 10 total; RTEC, ready-to-eat cereal.

With a few exceptions, the ranking of the top 10 sources of AS did not change across the AS intakes categories, and these top 10 sources provided 57‒74% and 51‒78% of daily AS in both all children and all adults, respectively (Table 6). However, for several sources (“soft drinks,” “fruit drinks,” and “tea”), the contribution to AS increased substantially across the AS intake categories in both children and adults. In all children, “high sugar RTEC” (>21.2 g/100 g) was the only top source of AS to decrease considerably (by ∼50%) across the AS intake categories. Meanwhile, in all adults, both “high sugar RTEC” and “cookies and brownies” decreased substantially (by 50% or more) across the AS intake categories (Table 6).

The top 10 sources of AS only contributed 8‒11% and 2‒7% of daily intakes of nutrients of public health concern (8% and 5% calcium; 9% and 2% vitamin D; 11% and 5% dietary fiber; and 9% and 7% potassium) in all children and all adults, respectively. When examining the contribution of the top 10 sources of AS to the intake of energy and nutrients of public health concern in all children, only “ice cream and frozen dairy desserts” and “high sugar RTEC” consistently provided >1% of >1 nutrient of public health concern to the diet across the AS intake categories. “Ice cream and frozen dairy desserts” provided ≥1% of calcium and potassium, whereas “high sugar RTEC” provided ≥1% of calcium, dietary fiber, and vitamin D. “High sugar RTEC” also provided 2 nutrients (dietary fiber and vitamin D) in multiples of its contribution to energy intakes (percentage of daily energy was 1.9‒2.6% and percentage of daily dietary fiber and vitamin D was 3.2‒5.7% and 5.8‒10.7%, respectively, across the AS categories). In all adults, none of the top 10 sources of AS provided ≥1% of >1 nutrient of public health concern, but “tea” consistently provided potassium, “ice cream and frozen desserts” consistently provided calcium, and “cookies and brownies” consistently provided dietary fiber across the AS intake categories (Table 7).

**TABLE 7:** Percent contribution of top 10 sources of added sugars, across added sugar intake categories, to energy and nutrient intakes among children and adults

Sources^1^	Calories from AS (AS intake categories)	% Contribution				
		Energy	Calcium	Fiber	Potassium	Vitamin D
**Children (aged 2‒18 y)**						
Soft drinks	<10% (AS10)	0.76	0.06	0.00	0.04	0.00
	10‒15% (AS10‒15)	2.04	0.18	0.00	0.11	0.00
	>15% (AS15)	4.92	0.48	0.00	0.31	0.00
Fruit drinks	<10% (AS10)	0.75	0.20	0.15	0.77	0.02
	10‒15% (AS10‒15)	1.80	0.65	0.41	1.89	0.10
	>15% (AS15)	3.46	0.87	0.58	3.61	0.00
Cookies and brownies	<10% (AS10)	2.56	0.28	1.39	0.70	0.04
	10‒15% (AS10‒15)	3.75	0.46	2.36	1.17	0.06
	>15% (AS15)	4.75	0.58	3.31	1.60	0.15
High sugar RTEC	<10% (AS10)	1.92	1.59	3.29	0.90	5.84
	10‒15% (AS10‒15)	2.43	2.21	4.97	1.32	8.15
	>15% (AS15)	2.61	2.61	5.65	1.35	10.71
Candy not containing chocolate	<10% (AS10)	0.56	0.04	0.04	0.12	0.00
	10‒15% (AS10‒15)	1.23	0.13	0.14	0.34	0.00
	>15% (AS15)	2.25	0.17	0.11	0.43	0.00
Ice cream and frozen dairy desserts	<10% (AS10)	1.44	1.36	0.61	1.08	0.30
	10‒15% (AS10‒15)	2.13	2.29	0.96	1.84	0.56
	>15% (AS15)	3.22	4.18	1.64	3.13	0.91
Tea	<10% (AS10)	0.22	0.03	0.03	0.27	0.01
	10‒15% (AS10‒15)	0.62	0.10	0.04	0.52	0.05
	>15% (AS15)	1.39	0.14	0.08	1.33	0.00
Cakes and pies	<10% (AS10)	0.55	0.15	0.30	0.20	0.09
	10‒15% (AS10‒15)	1.81	0.45	1.01	0.74	0.34
	>15% (AS15)	3.20	0.92	1.89	1.41	0.80
Jams, syrups, toppings	<10% (AS10)	0.41	0.06	0.14	0.10	0.00
	10‒15% (AS10‒15)	0.82	0.09	0.21	0.16	0.00
	>15% (AS15)	1.22	0.19	0.47	0.31	0.00
Doughnuts, sweet rolls, pastries	<10% (AS10)	1.68	0.52	0.94	0.37	0.04
	10‒15% (AS10‒15)	2.72	1.03	1.63	0.69	0.08
	>15% (AS15)	2.74	1.07	1.88	0.71	0.08
**Adults (aged 19+ y)**						
Soft drinks	<10% (AS10)	0.65	0.06	0.00	0.03	0.00
	10‒15% (AS10‒15)	2.45	0.24	0.00	0.14	0.00
	>15% (AS15)	8.34	0.84	0.00	0.62	0.00
Tea	<10% (AS10)	0.38	0.19	0.03	1.62	0.17
	10‒15% (AS10‒15)	0.83	0.24	0.04	1.68	0.16
	>15% (AS15)	2.85	0.44	0.12	3.02	0.30
Cakes and pies	<10% (AS10)	1.25	0.38	0.59	0.37	0.33
	10‒15% (AS10‒15)	2.93	0.81	1.45	0.96	0.79
	>15% (AS15)	3.71	1.32	2.20	1.41	1.24
Fruit drinks	<10% (AS10)	0.32	0.13	0.11	0.36	0.00
	10‒15% (AS10‒15)	0.97	0.36	0.34	0.93	0.01
	>15% (AS15)	1.80	0.63	0.45	1.93	0.03
Cookies and brownies	<10% (AS10)	1.93	0.28	1.04	0.50	0.05
	10‒15% (AS10‒15)	3.11	0.43	1.92	0.88	0.10
	>15% (AS15)	2.96	0.49	2.21	0.97	0.12
Sugars and honey	<10% (AS10)	0.33	0.02	0.00	0.02	0.00
	10‒15% (AS10‒15)	0.67	0.02	0.00	0.03	0.00
	>15% (AS15)	1.09	0.03	0.01	0.03	0.00
Ice cream and frozen dairy desserts	<10% (AS10)	1.18	1.46	0.55	0.82	0.36
	10‒15% (AS10‒15)	2.14	2.92	0.96	1.70	0.79
	>15% (AS15)	2.44	3.62	1.33	2.24	1.02
Candy containing chocolate	<10% (AS10)	0.71	0.35	0.54	0.35	0.01
	10‒15% (AS10‒15)	1.60	0.77	1.22	0.84	0.02
	>15% (AS15)	1.53	0.80	1.45	0.90	0.02
Sport and energy drinks	<10% (AS10)	0.14	0.02	0.00	0.05	0.00
	10‒15% (AS10‒15)	0.47	0.06	0.00	0.14	0.00
	>15% (AS15)	1.03	0.12	0.00	0.37	0.00
Candy not containing chocolate	<10% (AS10)	0.28	0.04	0.05	0.05	0.00
	10‒15% (AS10‒15)	0.54	0.08	0.10	0.11	0.00
	>15% (AS15)	1.17	0.15	0.21	0.22	0.00

Food sources were summed by subject and then multiplied day 1 sample weights to obtain a population weighted total intake of energy or nutrients; percentage contribution for each food category was calculated as the individual food category divided by the total intake.

Abbreviations: AS, added sugar; AS10, <10% calories from added sugar; AS10‒15, 10‒15% calories from added sugar; AS15, >15% calories from added sugar; RTEC, ready-to-eat cereal.

1 These are the top sources of AS in the general population of children and adults (not stratified by AS intake category).

“Higher sugar RTEC” and “lower sugar RTEC” (≤21.2 g/100 g) ranked as the fourth and twenty-seventh source, respectively, of AS and provided 5.44% and 0.67% (0.92 tsp/d and 0.12 tsp/d) of daily AS intake, respectively, among children aged 2‒18 y. Among adults age 19+ y, higher and lower sugar RTEC ranked as the twelfth and twenty-second source of AS and provided 2.37% and 0.73% (0.41 tsp/d and 0.13 tsp/d) of daily AS intake (Table 8). When we totaled contributions of higher sugar (>21.2 g/100 g) and lower sugar (≤21.2 g/100 g) RTEC to assess total RTEC contributions to the diet, we found AS from total RTEC provided 6.11% and 3.10% AS in the diet in children and adults, respectively (Table 8), ranking as the fourth and ninth source, respectively. Total RTEC contribution to AS decreased across the AS categories in children (10.0%, 6.9%, and 4.5%) and in adults (5.3%, 3.6%, and 2.0%). Total RTEC provided 2.7%, 6.4%, 1.7%, and 9.9% of calcium, dietary fiber, potassium and vitamin D, respectively to the diets of children and provided 1.5%, 5.2%, 1.4%, and 6.4% of calcium, dietary fiber, potassium and vitamin D, respectively to the diets of adults while only providing 3.1% of calories in children and 1.9% of calories in adults.

**TABLE 8:** Percent contribution of ready-to-eat cereal to total daily calories and nutrients.

Nutrients	Higher sugar (>21.2 g/100 g) RTEC				Lower sugar (≤21.2 g/100 g) RTEC				Total RTEC			
	All	Calories from AS (AS categories)			All	Calories from AS (AS intake categories)			All	Calories from AS (AS intake categories)		
		>10% (AS10)	10-15% AS10‒15	>15% (AS15)		<10% (AS10)	10-15% (AS10‒15)	>15% (AS15)		>10% (AS10)	10-15% (AS10‒15)	>15% (AS15)
**Children (aged 2‒18 y)**												
Calories	2.34	1.92	2.43	2.61	0.71	0.95	0.79	0.42	3.05	2.87	3.22	3.03
AS	5.44	8.37	6.11	4.21	0.67	1.64	0.82	0.30	6.11	10.00	6.93	4.51
Calcium	2.12	1.59	2.21	2.61	0.55	0.77	0.52	0.33	2.67	2.36	2.73	2.94
Dietary Fiber	4.60	3.29	4.97	5.65	1.78	2.09	1.99	1.22	6.38	5.38	6.96	6.87
Potassium	1.19	0.90	1.32	1.35	0.54	0.67	0.61	0.34	1.73	1.58	1.93	1.68
Vitamin D	8.11	5.84	8.15	10.71	1.79	2.04	2.02	1.24	9.90	7.88	10.17	11.96
**Adults (aged 19+ y)**												
Calories	1.11	0.96	1.25	1.23	0.78	0.90	0.84	0.56	1.89	1.86	2.08	1.79
AS	2.37	3.70	2.74	1.66	0.73	1.58	0.81	0.34	3.09	5.28	3.55	2.00
Calcium	0.85	0.60	1.19	0.98	0.68	0.76	0.65	0.55	1.53	1.36	1.84	1.54
Dietary Fiber	2.67	2.31	2.77	3.37	2.55	2.70	2.92	1.85	5.22	5.01	5.69	5.22
Potassium	0.76	0.67	0.81	0.89	0.63	0.69	0.68	0.48	1.40	1.36	1.50	1.37
Vitamin D	4.39	2.85	5.31	6.53	2.01	2.01	2.07	1.97	6.41	4.85	7.38	8.50

Food sources were summed by subject and then multiplied day 1 sample weights to obtain a population weighted total intake of AS; percentage contribution for each food category was calculated as the individual food category divided by the total intake.

Abbreviations: AS, added sugar; AS10, <10% calories from added sugar; AS10‒15, 10‒15% calories from added sugar; AS15, >15% calories from added sugar; RTEC, ready-to-eat cereal.

## Discussion

The results of the current analysis, the first study to comprehensively document nutrient intakes, nutrient adequacy, diet quality, and top sources of AS across the range of AS intakes in the United States population, indicate that diet quality, nutrient intake and percentage less than EAR increased or percentage greater than AI decreased with increasing percentage of calories from AS in the diets of children and adults. “Soft drinks,” “cookies and brownies,” “ice creams and frozen desserts,” “tea,” and “candies (containing chocolate or not)” were always among the top 10 sources of AS irrespective of AS category in both all children and all adults. We hypothesized that flavored dairy would be a top source of AS. However, we did not see nutrient-dense flavored dairy sources of AS, such as yogurt or milk, as a top source of AS for kids or adults. As dairy is an under-consumed food group, this analysis would suggest that flavored dairy is not a top source of AS.

Overall, sources of AS did not differ much in rank across the AS categories, but for certain sources (“soft drinks,” “fruit drinks,” and “tea”) the percentage contribution increased considerably as percentage of calories from AS increased, whereas 2 sources (“higher sugar RTEC” and “cookies and brownies”) decreased considerably as percentage of calories from AS increased. These changes in sources impacted diet quality scores, nutrient intakes, and percentage less than EAR or greater than AI. One of the key issues with ranking sources of AS and other items is the grouping of foods. Some research teams have grouped cookies and brownies, cakes and pies, and doughnuts, sweet rolls, and pastries as a single food group called sweet bakery products [6]. We, on the contrary, had these items as separate food categories; we actually used all 168 categories in the WWEIA to establish our AS listing. Thus, depending on the grouping of foods, the ranking of AS sources could change, as would the top 10 sources.

Although the top 2 sources of AS (“soft drinks” and “fruit drinks”) in our study provided <1% of any nutrients of public health concern, some of the other AS sources did provide ≥1% public health nutrients, and only “ice cream and frozen dairy desserts” and “high sugar RTEC” provided ≥1% of >1 of the public health nutrients. This suggests that some AS sources make a considerable contribution to nutrient intakes and thus removing these foods based on AS content may have unintended consequences.

We estimated that the average intake of AS is slightly over 13% and 12% calories for all children and all adults, respectively. This mean intake is similar to earlier estimates of AS intakes by us and others [5,10,22,23]. There has been a decline in AS intake among United States children age 2‒18 y from 17.3% daily calories in 2001‒2002 to 13.6% daily calories in 2017‒2018, primarily from reduced sugar-sweetened beverage consumption [8]. Median intake of AS for children and adults in our study was 12.5% and 10.5% of calories, respectively. Although both mean and median intake estimates exceed the DGA recommendation of AS10 [2], our data suggest that nearly a third of children and half of the adult population meet the recommendation. However, the data also suggest that nearly two-thirds of children and half of the adult population have AS intakes above the recommendation. We grouped individuals into 3 AS intake categories: *1*) AS10, which aligns with DGA; *2*) AS10‒15; and *3*) AS15, which exceeds the DGA recommendations. Examining diet quality across these AS intake categories, our data show that the HEI 2020 scores for both children and adults decreased with increasing calories from AS. HEI is a validated measure of diet quality to assess the dietary compliance/adherence to DGA recommendations [2] and has 13 components, each of which relates to the key recommendations [17,18].

Several studies have examined the relationship between total HEI 2015 scores, which are very similar to HEI 2020, and all-cause mortality in adults. In a pooled analysis of the Nurses’ Health Study and the Health Professionals Follow-Up Study, there was a 19% reduction in all-cause mortality comparing the highest (79 points) to the lowest (56 points) quintile of scores [24]. Panizza et al. [25] reported that there was a 21% reduction in all-cause mortality comparing the highest (80 points) to the lowest quintile (51 points) of HEI scores. Additionally, differences between the first and second quintiles of HEI 2015 scores were 8.7 points, which was associated with a 7% reduction in all-cause mortality. Thus, it is likely that the changes we saw in lower HEI scores in the AS15 group (∼12 points less than the AS10 group) may be associated with increased disease risk. Limited studies are available regarding the relationship of HEI scores to disease risk in healthy children; however, 1 study reported that in a low-income, multiethnic population of children (mean age of 9.3 y), a 10-point increase in total HEI 2020 score was associated with a small by significant 0.4% decrease in body fat percentage [26]. Others have shown an association of HEI scores on specific conditions like asthma [27] and metabolic-associated fatty liver disease [28].

Our observation of decreasing HEI 2020 scores indicates that the diet quality and dietary compliance with DGA recommendations decrease with increasing calories from AS. In addition to decreased HEI 2020 scores, intake of fruits and vegetables, whole grains, dairy, and protein foods also decreased with an increase in calories from AS. These food groups are important sources of many nutrients, including those that are considered of public health concern (calcium, fiber, potassium, and vitamin D) because their current intakes are low enough to impact one’s health [2]. Lower intake of these important food groups in the higher AS intake groups is expected to result in lower intakes of nutrients. In our analysis of NHANES 2011‒2018 data, we found that nutrient intakes and the proportion of the population not meeting the nutrient intake recommendations for fiber, calcium, potassium, and vitamin D, and numerous other nutrients, were gradually decreased with increasing calories from AS. Similar observations were also reported in earlier cross-sectional studies from both the United States and international cohorts [10,15,[29], [30], [31]].

Although some of the AS sources did provide ≥1% of public health nutrients, only “ice cream and frozen dairy desserts” and “high sugar RTEC” provided ≥1% for >1 of the public health nutrients. When we combined “high sugar RTEC” and “low sugar RTEC” (USDA separates RTEC into these WWEIA categories) to assess the total contribution of RTEC to the diet, we found that total RTEC provided public health nutrients at a percentage higher than it contributed to calories, underscoring the nutrient-dense nature of this food category.

RTEC are commonly consumed grain products rich in many vitamins and minerals, with many also providing whole grains and dietary fiber, and their frequent consumption, especially among children, is associated with higher diet quality, better nutrient adequacy, and lower BMI (in kg/m^2^) [20,[32], [33], [34]]. Our results also indicate that RTEC contributes significantly to the daily intake of several key nutrients. Although RTEC, especially the higher sugar type, is also among the top sources of AS (fourth in children, twelfth in adults) and contributes >5% and >2% of daily AS intakes among all children and all adults overall, it is worth noting that the contribution of RTEC to AS decreased by 50% going from AS10 to AS15, indicating that its relative contribution to AS intake is lower among those who consume the greatest percentage of calories from AS. In contrast, for several sources (“soft drinks,” “fruit drinks,” and “tea”), the contribution to AS increased substantially across the AS intake categories in both children and adults.

Although in general the differences seen between groups in AS10 compared with AS15 were similar across age groups, especially for food group intakes and HEI total and subcomponent scores, those 2‒4 y and those 60+ y had fewer nutrients that were adversely impacted by higher calories from AS. In the younger group, this may be due to parents ensuring nutrient-dense foods are provided to their children, even though some may contain AS. Although the same concept may apply in those 60+ y, the nutrients not impacted by higher calories from AS appear to be specifically associated with fortified grain products, which can also provide AS.

The present study has several strengths. The findings are generalizable to the United States population, due to the use of a nationally representative population database. We categorized individuals based on their AS intake, ranging from meeting dietary guideline recommendations to exceeding recommendations by 50% or more, going from AS10 to AS15. This allowed us to examine the association of AS intake, diet quality, nutrient intakes, and nutrient adequacy across the continuum of observed intakes in the United States population, as well as top sources of AS across AS intake categories. Additionally, to minimize misclassification errors, we used a 2-d mean intake, rather than a 1-d, to define intakes. The limitations of our study include the use of self-reported dietary intake data, which rely on memory and are subject to potential reporting bias and may under- or overestimate the actual intake. Additionally, NHANES is a cross-sectional survey, and therefore causality of sugar intake with nutrient intake cannot be inferred.

In conclusion, we found that higher AS intakes were associated with lower diet quality, lower nutrient intakes, and lower nutrient adequacy in both children and adults. Overall, sources of AS did not differ much in rank across the AS categories, but for certain sources (“soft drinks,” “fruit drinks,” and “tea”) the percentage contribution increased considerably as percentage of calories from AS increased, whereas 2 sources (“higher sugar RTEC” and “cookies and brownies”) decreased considerably as percentage of calories from AS increased. These changes in sources impacted diet quality scores, nutrient intakes, and nutrient adequacy. Public health efforts to decrease AS intake often focus on reducing intake across all food and beverage categories, in the population as a whole [35]. However, the evidence presented in this manuscript suggests that such efforts should take into consideration differences in food and beverage sources of AS and their nutrient density across the range of AS intake. Specifically, the analyses presented here underscore the important contributions of nutrient-dense sources of AS, like RTEC, to nutrient and whole-grain intakes. Flavored dairy is not among the top sources of AS, suggesting that discouraging intakes would have minimal impact on population intakes of AS. However, like RTEC, flavored dairy contributes meaningful amounts of under-consumed nutrients [36]. Overall, the findings presented in this manuscript can provide useful insights for policy and regulatory development related to nutrition and public health. For example, successful public health nutrition strategies to reduce AS could focus on reducing AS from non-nutrient-dense sources, especially among those whose AS intakes greatly exceed recommendations, although recognizing the contributions of nutrient-dense foods and beverages in the population as a whole.

## Author contributions

The authors’ responsibilities were as follows– MT, BG-J, VLF: participated in the formulation of the research question, design of the analyses, interpretation of the data, drafting of the manuscript, revision of the manuscript, and the approval of the final version; VLF: conducted data analysis; All authors are responsible for all aspects of the manuscript; and all authors: read and approved the final manuscript.

## Data availability

The datasets analyzed in this study are available in the Centers for Disease Control and Prevention repository; available online: http://www.cdc.gov/nchs/nhanes/. Upon request, authors are willing to help others to replicate or conduct similar analyses.

## Funding

Funding for this research was provided by the Bell Institute of Health and Nutrition, General Mills, Inc.

## Conflict of interest

MT and BG-J are employees of General Mills, Inc. VLF, at Nutrition Impact, LLC, performs consulting and database analyses for various food and beverage companies and related entities. He reports a relationship with the National Dairy Council that includes consulting or advisory activities and funding grants, and The Sugar Association, Inc, that includes funding grants.
