# Supplementary material for: Added Sugar Sources and Their Impact on Diet Quality and Nutrient Intakes: An Analysis Across Added Sugar Intake Levels in United States Children and Adults
Source: Curr Dev Nutr. 2025 Oct 3;9(11):107566. doi: 10.1016/j.cdnut.2025.107566 (PMC12605293; doi:10.1016/j.cdnut.2025.107566)
Supplement: multimedia component 1 [file mmc1.docx]

Supplemental Table 1. Intakes (2 days average) for food groups by % of calorie from added sugars (<10%, 10-15%, and >15%) in children and adults by age groups - NHANES 2011-2018 data, gender combined population.

|  | Calories from Added Sugar (Added Sugars Intake Categories) | | | | | | P_Linear Trend_ |
| --- | --- | --- | --- | --- | --- | --- | --- |
|  | <10%  (AS10) | | 10% to 15% (AS10-15) | | >15%  (AS15) | |  |
|  | Mean | SE | Mean | SE | Mean | SE |  |
| Children (aged 2-4 years) | | | | | | | |
| Added sugars (tsp eq) | 5.91 | 0.15 | 11.2 | 0.2 | 17.7 | 0.5 | <0.0001 |
| Total dairy (cup eq) | 2.18 | 0.07 | 2.04 | 0.07 | 1.79 | 0.11 | 0.0081 |
| Total fruits (cup eq) | 1.69 | 0.07 | 1.39 | 0.07 | 1.11 | 0.07 | <0.0001 |
| Total grain (oz eq) | 4.86 | 0.13 | 4.90 | 0.11 | 4.32 | 0.10 | 0.0048 |
| Whole grain (oz eq) | 0.82 | 0.04 | 0.72 | 0.05 | 0.58 | 0.06 | <0.0001 |
| Total protein foods (oz eq) | 3.01 | 0.08 | 2.81 | 0.09 | 2.69 | 0.16 | 0.0052 |
| Total vegetables* (cup eq) | 0.71 | 0.03 | 0.60 | 0.03 | 0.57 | 0.04 | 0.0001 |
| Children (aged 5-8 years) | | | | | | | |
| Added sugars (tsp eq) | 7.91 | 0.18 | 14.01 | 0.26 | 22.15 | 0.45 | <0.0001 |
| Total dairy (cup eq) | 2.25 | 0.08 | 2.18 | 0.05 | 1.91 | 0.06 | <0.0001 |
| Total fruits (cup eq) | 1.50 | 0.06 | 1.28 | 0.06 | 0.92 | 0.05 | <0.0001 |
| Total grain (oz eq) | 6.76 | 0.15 | 6.68 | 0.15 | 5.92 | 0.07 | <0.0001 |
| Whole grain (oz eq) | 1.05 | 0.05 | 0.96 | 0.05 | 0.67 | 0.03 | <0.0001 |
| Total protein foods (oz eq) | 3.92 | 0.12 | 3.71 | 0.11 | 3.19 | 0.10 | 0.0002 |
| Total vegetables* (cup eq) | 0.92 | 0.03 | 0.78 | 0.03 | 0.69 | 0.03 | <0.0001 |
| Children (aged 9-13 years) | | | | | | | |
| Added sugars (tsp eq) | 8.28 | 0.25 | 15.6 | 0.3 | 24.5 | 0.46 | <0.0001 |
| Total dairy (cup eq) | 2.20 | 0.10 | 2.12 | 0.06 | 1.79 | 0.05 | <0.0001 |
| Total fruits (cup eq) | 1.21 | 0.07 | 1.13 | 0.06 | 0.79 | 0.05 | <0.0001 |
| Total grain (oz eq) | 7.35 | 0.17 | 7.51 | 0.19 | 6.70 | 0.11 | 0.0002 |
| Whole grain (oz eq) | 1.02 | 0.06 | 1.02 | 0.05 | 0.76 | 0.04 | <0.0001 |
| Total protein foods (oz eq) | 4.48 | 0.15 | 4.43 | 0.16 | 3.93 | 0.08 | 0.0006 |
| Total vegetables* (cup eq) | 1.04 | 0.05 | 0.99 | 0.04 | 0.81 | 0.02 | <0.0001 |
| Children (aged 14-18 years) | | | | | | | |
| Added sugars (tsp eq) | 8.21 | 0.22 | 15.7 | 0.3 | 27.0 | 0.9 | <0.0001 |
| Total dairy (cup eq) | 2.11 | 0.11 | 1.92 | 0.07 | 1.69 | 0.08 | <0.0001 |
| Total fruits (cup eq) | 1.10 | 0.07 | 0.91 | 0.06 | 0.66 | 0.06 | <0.0001 |
| Total grain (oz eq) | 7.51 | 0.23 | 7.58 | 0.21 | 6.54 | 0.17 | <0.0001 |
| Whole grain (oz eq) | 1.00 | 0.05 | 0.88 | 0.05 | 0.70 | 0.06 | <0.0001 |
| Total protein foods (oz eq) | 6.02 | 0.34 | 5.08 | 0.20 | 4.41 | 0.14 | <0.0001 |
| Total vegetables* (cup eq) | 1.17 | 0.06 | 0.99 | 0.03 | 0.93 | 0.03 | <0.0001 |
| Adults (aged 19-30 years) | | | | | | | |
| Added sugars (tsp eq) | 8.05 | 0.22 | 17.58 | 0.30 | 30.76 | 0.56 | <0.0001 |
| Total dairy (cup eq) | 1.75 | 0.05 | 1.85 | 0.10 | 1.56 | 0.05 | 0.0001 |
| Total fruits (cup eq) | 1.13 | 0.06 | 0.90 | 0.05 | 0.50 | 0.03 | 0.0000 |
| Total grain (oz eq) | 7.08 | 0.16 | 7.70 | 0.20 | 6.71 | 0.15 | 0.0029 |
| Whole grain (oz eq) | 0.96 | 0.06 | 0.87 | 0.06 | 0.53 | 0.03 | <0.0001 |
| Total protein foods (oz eq) | 7.18 | 0.19 | 6.21 | 0.16 | 5.33 | 0.13 | <0.0001 |
| Total vegetables* (cup eq) | 1.61 | 0.05 | 1.34 | 0.04 | 1.12 | 0.04 | <0.0001 |
| Adults (aged 31-59 years) | | | | | | | |
| Added sugars (tsp eq) | 7.52 | 0.12 | 16.84 | 0.23 | 31.60 | 0.56 | <0.0001 |
| Total dairy (cup eq) | 1.51 | 0.04 | 1.66 | 0.05 | 1.49 | 0.05 | 0.0896 |
| Total fruits (cup eq) | 1.07 | 0.03 | 0.95 | 0.04 | 0.68 | 0.04 | <0.0001 |
| Total grain (oz eq) | 6.57 | 0.11 | 6.85 | 0.12 | 6.29 | 0.13 | 0.0005 |
| Whole grain (oz eq) | 1.09 | 0.04 | 1.00 | 0.05 | 0.64 | 0.03 | <0.0001 |
| Total protein foods (oz eq) | 6.87 | 0.11 | 6.35 | 0.10 | 5.71 | 0.10 | <0.0001 |
| Total vegetables* (cup eq) | 1.80 | 0.03 | 1.57 | 0.03 | 1.30 | 0.03 | <0.0001 |
| Adults (aged 60+ years) | | | | | | | |
| Added sugars (tsp eq) | 7.33 | 0.14 | 15.23 | 0.21 | 26.02 | 0.55 | <0.0001 |
| Total dairy (cup eq) | 1.41 | 0.04 | 1.44 | 0.05 | 1.29 | 0.05 | 0.0581 |
| Total fruits (cup eq) | 1.21 | 0.04 | 1.03 | 0.04 | 0.86 | 0.04 | <0.0001 |
| Total grain (oz eq) | 5.79 | 0.11 | 5.99 | 0.10 | 5.48 | 0.13 | 0.1965 |
| Whole grain (oz eq) | 1.18 | 0.04 | 1.09 | 0.05 | 0.86 | 0.05 | <0.0001 |
| Total protein foods (oz eq) | 6.20 | 0.10 | 5.87 | 0.12 | 4.74 | 0.10 | <0.0001 |
| Total vegetables* (cup eq) | 1.74 | 0.04 | 1.55 | 0.05 | 1.24 | 0.04 | <0.0001 |

Regression analysis was conducted with added sugars as percentage of calories as a continuous variable determined as the average of two days of intake adjusting for the complex sample design of NHANES and using 2-day dietary weights. *Total vegetables exclude legumes

Supplemental Table 2. Healthy Eating Index (2 days average) by % of calories from added sugars in children and adults by age groups - NHANES 2011-2018 data, gender combined population.

|  | Calories from Added Sugar (Added Sugars Intake Categories) | | | | | | | P_Linear Trend_ |
| --- | --- | --- | --- | --- | --- | --- | --- | --- |
|  | <10%  (AS10) | | 10% to 15% (AS10-15) | | >15%  (AS15) | | |  |
|  | Mean | SE | Mean | SE | Mean | SE |  | |
| Children (aged 2-4 years) | | | | | | | | |
| HEI-2020 total score | 59.6 | 0.6 | 56.0 | 0.8 | 52.5 | 0.9 | <0.0001 | |
| Component score for total fruit | 4.23 | 0.07 | 3.95 | 0.08 | 3.44 | 0.12 | <0.0001 | |
| Component score for whole fruit | 4.06 | 0.08 | 3.78 | 0.10 | 3.20 | 0.16 | 0.0001 | |
| Component score for total vegetables | 2.39 | 0.09 | 1.91 | 0.09 | 1.82 | 0.10 | <0.0001 | |
| Component score for greens and beans | 1.61 | 0.11 | 1.16 | 0.11 | 0.84 | 0.10 | <0.0001 | |
| Component score for whole grains | 3.64 | 0.16 | 3.27 | 0.19 | 2.77 | 0.26 | 0.0006 | |
| Component score for dairy | 8.52 | 0.13 | 8.33 | 0.17 | 7.48 | 0.25 | 0.0031 | |
| Component score for total protein foods | 3.83 | 0.06 | 3.66 | 0.10 | 3.34 | 0.11 | <0.0001 | |
| Component score for seafood and plant protein | 2.31 | 0.10 | 2.13 | 0.16 | 1.86 | 0.18 | 0.0033 | |
| Component score for fatty acid ratio | 3.25 | 0.17 | 3.26 | 0.23 | 3.81 | 0.24 | 0.1749 | |
| Component score for refined grain | 5.96 | 0.20 | 5.92 | 0.16 | 7.01 | 0.20 | 0.0001 | |
| Component score for sodium | 5.39 | 0.16 | 6.18 | 0.18 | 6.76 | 0.19 | <0.0001 | |
| Component score for saturated fat | 4.94 | 0.20 | 5.32 | 0.19 | 6.53 | 0.19 | <0.0001 | |
| Component score for added sugar | 9.50 | 0.03 | 7.14 | 0.05 | 3.69 | 0.14 | <0.0001 | |
| Children (aged 5-8 years) | | | | | | | | |
| HEI-2020 total score | 56.4 | 0.6 | 52.6 | 0.5 | 47.2 | 0.5 | <0.0001 | |
| Component score for total fruit | 3.81 | 0.10 | 3.47 | 0.09 | 2.66 | 0.12 | <0.0001 | |
| Component score for whole fruit | 3.72 | 0.11 | 3.43 | 0.11 | 2.70 | 0.11 | <0.0001 | |
| Component score for total vegetables | 2.52 | 0.09 | 2.03 | 0.08 | 1.84 | 0.06 | <0.0001 | |
| Component score for greens and beans | 1.57 | 0.11 | 1.12 | 0.09 | 0.91 | 0.08 | <0.0001 | |
| Component score for whole grains | 3.88 | 0.19 | 3.55 | 0.17 | 2.47 | 0.12 | <0.0001 | |
| Component score for dairy | 8.15 | 0.13 | 7.95 | 0.12 | 7.24 | 0.16 | <0.0001 | |
| Component score for total protein foods | 4.10 | 0.06 | 3.87 | 0.06 | 3.46 | 0.08 | <0.0001 | |
| Component score for seafood and plant protein | 2.56 | 0.12 | 2.29 | 0.11 | 1.75 | 0.09 | <0.0001 | |
| Component score for fatty acid ratio | 3.37 | 0.18 | 3.30 | 0.14 | 3.44 | 0.17 | 0.9439 | |
| Component score for refined grain | 4.43 | 0.23 | 4.74 | 0.18 | 5.50 | 0.17 | <0.0001 | |
| Component score for sodium | 4.16 | 0.16 | 4.89 | 0.18 | 6.22 | 0.19 | <0.0001 | |
| Component score for saturated fat | 4.76 | 0.25 | 4.96 | 0.15 | 5.70 | 0.12 | <0.0001 | |
| Component score for added sugar | 9.32 | 0.05 | 6.99 | 0.05 | 3.36 | 0.10 | <0.0001 | |
| Children (aged 9-13 years) | | | | | | | | |
| HEI-2020 total score | 53.5 | 0.6 | 50.0 | 0.5 | 45.2 | 0.7 | <0.0001 | |
| Component score for total fruit | 3.22 | 0.12 | 2.84 | 0.10 | 2.19 | 0.10 | <0.0001 | |
| Component score for whole fruit | 3.22 | 0.13 | 2.90 | 0.12 | 2.28 | 0.11 | <0.0001 | |
| Component score for total vegetables | 2.71 | 0.08 | 2.40 | 0.07 | 2.00 | 0.06 | <0.0001 | |
| Component score for greens and beans | 1.70 | 0.11 | 1.37 | 0.10 | 1.10 | 0.09 | <0.0001 | |
| Component score for whole grains | 3.57 | 0.20 | 3.28 | 0.16 | 2.55 | 0.13 | <0.0001 | |
| Component score for dairy | 7.57 | 0.16 | 7.17 | 0.14 | 6.51 | 0.14 | <0.0001 | |
| Component score for total protein foods | 4.23 | 0.05 | 3.95 | 0.09 | 3.69 | 0.06 | <0.0001 | |
| Component score for seafood and plant protein | 2.39 | 0.11 | 1.90 | 0.11 | 1.88 | 0.10 | 0.0001 | |
| Component score for fatty acid ratio | 3.85 | 0.22 | 3.62 | 0.14 | 3.64 | 0.13 | 0.5092 | |
| Component score for refined grain | 3.83 | 0.16 | 4.45 | 0.18 | 5.10 | 0.15 | <0.0001 | |
| Component score for sodium | 3.15 | 0.15 | 4.28 | 0.15 | 5.35 | 0.15 | <0.0001 | |
| Component score for saturated fat | 4.69 | 0.18 | 4.94 | 0.16 | 5.63 | 0.15 | <0.0001 | |
| Component score for added sugar | 9.32 | 0.03 | 6.93 | 0.04 | 3.26 | 0.07 | <0.0001 | |
| Children (aged 14-18 years) | | | | | | | | |
| HEI-2020 total score | 52.6 | 0.8 | 48.0 | 0.7 | 44.2 | 0.5 | <0.0001 | |
| Component score for total fruit | 2.70 | 0.10 | 2.29 | 0.12 | 1.74 | 0.12 | <0.0001 | |
| Component score for whole fruit | 2.69 | 0.11 | 2.17 | 0.10 | 1.67 | 0.12 | <0.0001 | |
| Component score for total vegetables | 2.77 | 0.08 | 2.40 | 0.08 | 2.19 | 0.05 | <0.0001 | |
| Component score for greens and beans | 1.66 | 0.13 | 1.18 | 0.11 | 1.03 | 0.10 | <0.0001 | |
| Component score for whole grains | 3.36 | 0.16 | 2.88 | 0.15 | 2.34 | 0.17 | <0.0001 | |
| Component score for dairy | 6.94 | 0.19 | 6.62 | 0.16 | 5.95 | 0.18 | <0.0001 | |
| Component score for total protein foods | 4.47 | 0.05 | 4.12 | 0.08 | 3.89 | 0.07 | <0.0001 | |
| Component score for seafood and plant protein | 2.46 | 0.12 | 2.18 | 0.11 | 1.71 | 0.08 | <0.0001 | |
| Component score for fatty acid ratio | 4.53 | 0.23 | 4.07 | 0.23 | 4.03 | 0.15 | 0.1671 | |
| Component score for refined grain | 4.18 | 0.18 | 4.24 | 0.22 | 5.63 | 0.15 | <0.0001 | |
| Component score for sodium | 2.45 | 0.14 | 3.79 | 0.18 | 5.05 | 0.18 | <0.0001 | |
| Component score for saturated fat | 4.96 | 0.24 | 5.09 | 0.18 | 6.05 | 0.15 | <0.0001 | |
| Component score for added sugar | 9.42 | 0.03 | 6.94 | 0.05 | 2.90 | 0.07 | <0.0001 | |
| Adults (aged 19-30 years) | | | | | | | | |
| HEI-2020 total score | 55.1 | 0.7 | 49.9 | 0.7 | 43.3 | 0.5 | <0.0001 | |
| Component score for total fruit | 2.37 | 0.10 | 2.10 | 0.10 | 1.30 | 0.07 | <0.0001 | |
| Component score for whole fruit | 2.40 | 0.11 | 2.04 | 0.14 | 1.26 | 0.08 | <0.0001 | |
| Component score for total vegetables | 3.32 | 0.06 | 2.82 | 0.07 | 2.45 | 0.06 | <0.0001 | |
| Component score for greens and beans | 2.16 | 0.09 | 1.70 | 0.11 | 1.27 | 0.08 | <0.0001 | |
| Component score for whole grains | 2.89 | 0.16 | 2.46 | 0.16 | 1.54 | 0.07 | <0.0001 | |
| Component score for dairy | 5.80 | 0.11 | 5.81 | 0.22 | 5.19 | 0.10 | <0.0001 | |
| Component score for total protein foods | 4.59 | 0.04 | 4.40 | 0.05 | 4.14 | 0.05 | <0.0001 | |
| Component score for seafood and plant protein | 3.02 | 0.10 | 2.54 | 0.13 | 1.98 | 0.08 | <0.0001 | |
| Component score for fatty acid ratio | 5.00 | 0.13 | 4.50 | 0.14 | 4.43 | 0.14 | 0.0152 | |
| Component score for refined grain | 5.39 | 0.14 | 5.30 | 0.18 | 5.83 | 0.13 | 0.0003 | |
| Component score for sodium | 2.99 | 0.15 | 3.76 | 0.16 | 4.94 | 0.15 | 0.0000 | |
| Component score for saturated fat | 5.57 | 0.15 | 5.45 | 0.15 | 6.38 | 0.12 | 0.0000 | |
| Component score for added sugar | 9.56 | 0.02 | 7.01 | 0.04 | 2.57 | 0.08 | 0.0000 | |
| Adults (aged 31-59 years) | | | | | | | | |
| HEI-2020 total score | 58.3 | 0.4 | 53.8 | 0.5 | 46.8 | 0.4 | <0.0001 | |
| Component score for total fruit | 2.55 | 0.06 | 2.28 | 0.07 | 1.64 | 0.05 | <0.0001 | |
| Component score for whole fruit | 2.81 | 0.06 | 2.51 | 0.09 | 1.78 | 0.06 | <0.0001 | |
| Component score for total vegetables | 3.70 | 0.03 | 3.27 | 0.04 | 2.73 | 0.05 | <0.0001 | |
| Component score for greens and beans | 2.61 | 0.06 | 2.17 | 0.08 | 1.54 | 0.06 | <0.0001 | |
| Component score for whole grains | 3.28 | 0.10 | 2.97 | 0.12 | 1.94 | 0.09 | <0.0001 | |
| Component score for dairy | 5.28 | 0.08 | 5.42 | 0.13 | 4.87 | 0.11 | <0.0001 | |
| Component score for total protein foods | 4.68 | 0.02 | 4.56 | 0.03 | 4.27 | 0.04 | <0.0001 | |
| Component score for seafood and plant protein | 3.35 | 0.06 | 3.02 | 0.07 | 2.49 | 0.07 | <0.0001 | |
| Component score for fatty acid ratio | 5.48 | 0.10 | 4.86 | 0.13 | 4.45 | 0.11 | <0.0001 | |
| Component score for refined grain | 6.06 | 0.10 | 6.16 | 0.12 | 6.80 | 0.11 | <0.0001 | |
| Component score for sodium | 3.14 | 0.08 | 3.93 | 0.13 | 5.36 | 0.10 | <0.0001 | |
| Component score for saturated fat | 5.71 | 0.08 | 5.66 | 0.11 | 6.23 | 0.09 | <0.0001 | |
| Component score for added sugar | 9.62 | 0.02 | 7.00 | 0.03 | 2.66 | 0.06 | <0.0001 | |
| Adults (aged 60+ years) | | | | | | | | |
| HEI-2020 total score | 60.5 | 0.5 | 56.2 | 0.7 | 50.0 | 0.4 | <0.0001 | |
| Component score for total fruit | 3.01 | 0.05 | 2.65 | 0.08 | 2.29 | 0.09 | <0.0001 | |
| Component score for whole fruit | 3.36 | 0.06 | 2.98 | 0.10 | 2.59 | 0.09 | <0.0001 | |
| Component score for total vegetables | 3.81 | 0.04 | 3.37 | 0.06 | 2.94 | 0.07 | <0.0001 | |
| Component score for greens and beans | 2.32 | 0.07 | 1.98 | 0.10 | 1.57 | 0.11 | <0.0001 | |
| Component score for whole grains | 3.94 | 0.13 | 3.48 | 0.14 | 2.75 | 0.12 | <0.0001 | |
| Component score for dairy | 5.39 | 0.10 | 5.25 | 0.12 | 4.79 | 0.13 | <0.0001 | |
| Component score for total protein foods | 4.70 | 0.02 | 4.57 | 0.03 | 4.25 | 0.04 | <0.0001 | |
| Component score for seafood and plant protein | 3.35 | 0.07 | 3.28 | 0.08 | 2.54 | 0.09 | <0.0001 | |
| Component score for fatty acid ratio | 5.49 | 0.13 | 5.04 | 0.17 | 4.33 | 0.11 | <0.0001 | |
| Component score for refined grain | 6.57 | 0.12 | 6.90 | 0.12 | 7.29 | 0.13 | <0.0001 | |
| Component score for sodium | 3.37 | 0.11 | 4.53 | 0.13 | 5.78 | 0.15 | <0.0001 | |
| Component score for saturated fat | 5.70 | 0.12 | 5.15 | 0.14 | 5.91 | 0.14 | 0.0229 | |
| Component score for added sugar | 9.52 | 0.01 | 7.08 | 0.03 | 2.99 | 0.10 | <0.0001 | |

Regression analysis was conducted with added sugars as percentage of calories as a continuous variable determined as the average of two days of intake adjusting for the complex sample design of NHANES and using 2-day dietary weights.

Supplemental Table 3. Nutrient intakes (2 days average) by % of calories from added sugars in children and adults by age groups - NHANES 2011-2018 data, gender combined population.

|  | Calories from Added Sugar (Added Sugars Intake Categories) | | | | | | P_Linear Trend_ |
| --- | --- | --- | --- | --- | --- | --- | --- |
|  | <10%  (AS10) | | 10% to 15% (AS10-15) | | >15%  (AS15) | |  |
|  | Mean | SE | Mean | SE | Mean | SE |  |
| Children (aged 2-4 years) | | | | | | | |
| Calcium (mg) | 988 | 24 | 931 | 22 | 831 | 31 | 0.0004 |
| Copper (mg) | 0.78 | 0.03 | 0.72 | 0.01 | 0.74 | 0.03 | 0.1559 |
| Iron (mg) | 10.7 | 0.3 | 11.2 | 0.3 | 10.1 | 0.3 | 0.3352 |
| Magnesium (mg) | 206 | 4 | 193 | 3 | 185 | 5 | 0.0014 |
| Phosphorus (mg) | 1105 | 21 | 1064 | 19 | 950 | 30 | 0.0003 |
| Selenium (µg) | 76.2 | 1.4 | 71.1 | 1.4 | 61.9 | 1.6 | <0.0001 |
| Zinc (mg) | 7.85 | 0.18 | 7.84 | 0.17 | 7.17 | 0.20 | 0.0499 |
| Vitamin A, RE (µg) | 592 | 23 | 577 | 16 | 480 | 21 | 0.0007 |
| Thiamin (mg) | 1.23 | 0.03 | 1.26 | 0.03 | 1.11 | 0.03 | 0.0261 |
| Riboflavin (mg) | 1.72 | 0.04 | 1.72 | 0.03 | 1.54 | 0.05 | 0.0540 |
| Niacin (mg) | 15.6 | 0.4 | 16.0 | 0.3 | 14.7 | 0.5 | 0.1832 |
| Folate, DFE (µg) | 406 | 12 | 421 | 12 | 362 | 16 | 0.0875 |
| Vitamin B6 (mg) | 1.45 | 0.04 | 1.42 | 0.03 | 1.30 | 0.05 | 0.0255 |
| Vitamin B12 (µg) | 4.22 | 0.19 | 4.01 | 0.10 | 3.59 | 0.11 | 0.0789 |
| Vitamin C (mg) | 83.6 | 4.4 | 72.6 | 3.5 | 82.5 | 5.2 | 0.8374 |
| Vitamin D (µg) | 6.37 | 0.17 | 5.83 | 0.17 | 5.26 | 0.32 | 0.0026 |
| Vitamin E, ATE (mg) | 5.51 | 0.17 | 5.50 | 0.14 | 5.32 | 0.19 | 0.4179 |
| Dietary fiber (g) | 12.7 | 0.3 | 11.5 | 0.2 | 10.3 | 0.3 | <0.0001 |
| Potassium (mg) | 2051 | 43 | 1904 | 37 | 1798 | 53 | 0.0004 |
| Sodium (mg) | 2176 | 42 | 2130 | 37 | 2043 | 55 | 0.0545 |
| Choline (mg) | 228 | 5 | 208 | 4 | 196 | 8 | 0.0007 |
| Children (aged 5-8 years) | | | | | | | |
| Calcium (mg) | 1070 | 26 | 1060 | 19 | 919 | 23 | <0.0001 |
| Copper (mg) | 0.93 | 0.02 | 0.89 | 0.02 | 0.81 | 0.02 | <0.0001 |
| Iron (mg) | 13.6 | 0.3 | 13.8 | 0.3 | 12.8 | 0.2 | 0.0198 |
| Magnesium (mg) | 241 | 4 | 229 | 4 | 204 | 3 | <0.0001 |
| Phosphorus (mg) | 1292 | 27 | 1250 | 20 | 1112 | 17 | <0.0001 |
| Selenium (µg) | 99.6 | 3.6 | 91.6 | 1.6 | 77.8 | 1.4 | <0.0001 |
| Zinc (mg) | 9.80 | 0.22 | 9.60 | 0.18 | 8.56 | 0.16 | <0.0001 |
| Vitamin A, RE (µg) | 649 | 18 | 637 | 18 | 579 | 20 | 0.0022 |
| Thiamin (mg) | 1.53 | 0.02 | 1.54 | 0.03 | 1.40 | 0.02 | <0.0001 |
| Riboflavin (mg) | 1.92 | 0.04 | 1.94 | 0.03 | 1.80 | 0.04 | 0.0032 |
| Niacin (mg) | 20.1 | 0.4 | 20.2 | 0.4 | 18.7 | 0.3 | 0.0009 |
| Folate, DFE (µg) | 512 | 11 | 519 | 15 | 470 | 13 | 0.0066 |
| Vitamin B6 (mg) | 1.69 | 0.03 | 1.65 | 0.03 | 1.53 | 0.03 | 0.0070 |
| Vitamin B12 (µg) | 4.46 | 0.14 | 4.67 | 0.12 | 4.27 | 0.11 | 0.1355 |
| Vitamin C (mg) | 81.1 | 3.5 | 76.6 | 3.0 | 70.8 | 3.2 | 0.0349 |
| Vitamin D (µg) | 5.85 | 0.24 | 5.99 | 0.17 | 5.21 | 0.18 | 0.0036 |
| Vitamin E, ATE (mg) | 6.86 | 0.18 | 6.87 | 0.18 | 6.50 | 0.17 | 0.0789 |
| Dietary fiber (g) | 15.6 | 0.3 | 14.3 | 0.3 | 12.0 | 0.2 | <0.0001 |
| Potassium (mg) | 2244 | 43 | 2132 | 35 | 1921 | 33 | <0.0001 |
| Sodium (mg) | 2856 | 61 | 2840 | 52 | 2575 | 43 | <0.0001 |
| Choline (mg) | 256 | 6 | 241 | 5 | 209 | 3 | <0.0001 |
| Children (aged 9-13 years) | | | | | | | |
| Calcium (mg) | 1078 | 32 | 1081 | 21 | 936 | 19 | <0.0001 |
| Copper (mg) | 0.96 | 0.03 | 1.05 | 0.06 | 0.90 | 0.02 | 0.0054 |
| Iron (mg) | 14.3 | 0.4 | 15.8 | 0.5 | 14.2 | 0.2 | 0.5403 |
| Magnesium (mg) | 248 | 6 | 249 | 5 | 222 | 4 | <0.0001 |
| Phosphorus (mg) | 1346 | 33 | 1355 | 24 | 1193 | 18 | <0.0001 |
| Selenium (µg) | 103 | 3 | 104 | 2 | 89.5 | 1.4 | <0.0001 |
| Zinc (mg) | 10.5 | 0.3 | 11.1 | 0.2 | 9.86 | 0.21 | 0.0204 |
| Vitamin A, RE (µg) | 623 | 23 | 691 | 37 | 569 | 15 | 0.0008 |
| Thiamin (mg) | 1.62 | 0.04 | 1.71 | 0.03 | 1.53 | 0.03 | 0.0177 |
| Riboflavin (mg) | 1.97 | 0.06 | 2.10 | 0.05 | 1.87 | 0.04 | 0.0403 |
| Niacin (mg) | 22.3 | 0.5 | 23.4 | 0.5 | 21.4 | 0.4 | 0.0602 |
| Folate, DFE (µg) | 529 | 15 | 586 | 21 | 527 | 14 | 0.5022 |
| Vitamin B6 (mg) | 1.80 | 0.06 | 1.88 | 0.04 | 1.69 | 0.03 | 0.0162 |
| Vitamin B12 (µg) | 4.86 | 0.21 | 5.31 | 0.38 | 4.50 | 0.13 | 0.0389 |
| Vitamin C (mg) | 73.4 | 3.6 | 77.8 | 3.0 | 65.3 | 2.1 | 0.0363 |
| Vitamin D (µg) | 5.81 | 0.27 | 5.55 | 0.18 | 4.71 | 0.17 | 0.0001 |
| Vitamin E, (mg) | 7.24 | 0.37 | 7.96 | 0.25 | 7.26 | 0.21 | 0.7702 |
| Dietary fiber (g) | 16.1 | 0.5 | 15.7 | 0.4 | 13.4 | 0.2 | <0.0001 |
| Potassium (mg) | 2290 | 62 | 2280 | 35 | 2018 | 32 | <0.0001 |
| Sodium (mg) | 3210 | 75 | 3263 | 68 | 2974 | 42 | 0.0017 |
| Choline (mg) | 269 | 8 | 269 | 6 | 234 | 5 | 0.0001 |
| Children (aged 14-18 years) | | | | | | | |
| Calcium (mg) | 1115 | 39 | 1001 | 25 | 896 | 31 | <0.0001 |
| Copper (mg) | 1.11 | 0.04 | 1.00 | 0.02 | 0.90 | 0.03 | <0.0001 |
| Iron (mg) | 16.1 | 0.7 | 15.2 | 0.4 | 13.8 | 0.5 | 0.0025 |
| Magnesium (mg) | 280 | 12 | 248 | 5 | 222 | 6 | <0.0001 |
| Phosphorus (mg) | 1464 | 49 | 1327 | 27 | 1194 | 31 | <0.0001 |
| Selenium (µg) | 122 | 3 | 110 | 2 | 94.7 | 2.4 | <0.0001 |
| Zinc (mg) | 12.0 | 0.7 | 10.5 | 0.3 | 9.87 | 0.37 | 0.0005 |
| Vitamin A, RE (µg) | 647 | 29 | 562 | 15 | 514 | 21 | <0.0001 |
| Thiamin (mg) | 1.77 | 0.06 | 1.68 | 0.04 | 1.53 | 0.06 | 0.0003 |
| Riboflavin (mg) | 2.17 | 0.12 | 1.97 | 0.06 | 1.87 | 0.08 | 0.0144 |
| Niacin (mg) | 26.8 | 1.1 | 24.9 | 0.6 | 24.5 | 1.0 | 0.4729 |
| Folate, DFE (µg) | 597 | 26 | 553 | 17 | 485 | 19 | <0.0001 |
| Vitamin B6 (mg) | 2.12 | 0.11 | 1.96 | 0.08 | 1.95 | 0.10 | 0.5660 |
| Vitamin B12 (µg) | 5.31 | 0.30 | 4.91 | 0.19 | 5.32 | 0.35 | 0.6349 |
| Vitamin C (mg) | 77.0 | 4.1 | 67.2 | 4.3 | 63.2 | 4.3 | 0.0010 |
| Vitamin D (µg) | 5.72 | 0.28 | 4.62 | 0.21 | 4.20 | 0.19 | <0.0001 |
| Vitamin E, ATE (mg) | 8.90 | 0.92 | 7.85 | 0.25 | 6.97 | 0.17 | 0.0158 |
| Dietary fiber (g) | 16.9 | 0.6 | 15.2 | 0.3 | 12.8 | 0.3 | <0.0001 |
| Potassium (mg) | 2445 | 75 | 2199 | 43 | 2048 | 55 | <0.0001 |
| Sodium (mg) | 3584 | 83 | 3382 | 63 | 3139 | 84 | 0.0056 |
| Choline (mg) | 315 | 12 | 267 | 7 | 241 | 6 | <0.0001 |
| Adults (aged 19-30 years) | | | | | | | |
| Calcium (mg) | 1082 | 22 | 1088 | 37 | 893 | 19 | <0.0001 |
| Copper (mg) | 1.33 | 0.03 | 1.24 | 0.04 | 0.98 | 0.02 | <0.0001 |
| Iron (mg) | 15.3 | 0.3 | 15.6 | 0.4 | 13.9 | 0.3 | <0.0001 |
| Magnesium (mg) | 331 | 6 | 303 | 8 | 243 | 5 | <0.0001 |
| Phosphorus (mg) | 1527 | 28 | 1484 | 32 | 1271 | 22 | <0.0001 |
| Selenium (µg) | 133 | 2 | 124 | 3 | 105 | 2 | <0.0001 |
| Zinc (mg) | 12.2 | 0.3 | 12.2 | 0.3 | 10.4 | 0.2 | <0.0001 |
| Vitamin A, RE (µg) | 669 | 18 | 630 | 26 | 495 | 15 | <0.0001 |
| Thiamin (mg) | 1.73 | 0.04 | 1.78 | 0.04 | 1.53 | 0.03 | <0.0001 |
| Riboflavin (mg) | 2.19 | 0.04 | 2.21 | 0.07 | 1.96 | 0.05 | <0.0001 |
| Niacin (mg) | 28.5 | 0.6 | 29.2 | 0.7 | 27.2 | 0.6 | 0.0202 |
| Folate, DFE (µg) | 585 | 15 | 601 | 19 | 508 | 16 | 0.0000 |
| Vitamin B6 (mg) | 2.33 | 0.06 | 2.39 | 0.09 | 2.22 | 0.08 | 0.0286 |
| Vitamin B12 (µg) | 5.10 | 0.15 | 5.54 | 0.24 | 5.23 | 0.15 | 0.9400 |
| Vitamin C (mg) | 85.3 | 3.0 | 77.5 | 3.0 | 62.5 | 2.6 | <0.0001 |
| Vitamin D (µg) | 5.12 | 0.23 | 4.74 | 0.28 | 3.59 | 0.13 | <0.0001 |
| Vitamin E, ATE (mg) | 9.93 | 0.29 | 9.39 | 0.36 | 7.66 | 0.21 | <0.0001 |
| Dietary fiber (g) | 18.6 | 0.5 | 17.2 | 0.4 | 13.3 | 0.3 | <0.0001 |
| Potassium (mg) | 2730 | 49 | 2565 | 50 | 2164 | 43 | <0.0001 |
| Sodium (mg) | 3752 | 67 | 3826 | 71 | 3444 | 59 | <0.0001 |
| Choline (mg) | 373 | 8 | 331 | 7 | 279 | 5 | <0.0001 |
| Adults (aged 31-59 years) | | | | | | | |
| Calcium (mg) | 989 | 13 | 1029 | 21 | 909 | 19 | <0.0001 |
| Copper (mg) | 1.36 | 0.02 | 1.25 | 0.02 | 1.11 | 0.02 | <0.0001 |
| Iron (mg) | 14.6 | 0.2 | 15.4 | 0.3 | 14.3 | 0.3 | 0.0151 |
| Magnesium (mg) | 339 | 4 | 315 | 5 | 268 | 4 | <0.0001 |
| Phosphorus (mg) | 1458 | 16 | 1438 | 20 | 1309 | 21 | <0.0001 |
| Selenium (µg) | 125 | 2 | 117 | 1 | 106 | 1 | <0.0001 |
| Zinc (mg) | 11.7 | 0.1 | 11.6 | 0.2 | 10.7 | 0.2 | <0.0001 |
| Vitamin A, RE (µg) | 673 | 16 | 660 | 16 | 572 | 15 | <0.0001 |
| Thiamin (mg) | 1.64 | 0.02 | 1.68 | 0.03 | 1.54 | 0.03 | <0.0001 |
| Riboflavin (mg) | 2.18 | 0.04 | 2.28 | 0.04 | 2.13 | 0.05 | 0.0375 |
| Niacin (mg) | 26.9 | 0.4 | 27.4 | 0.4 | 25.4 | 0.5 | 0.0018 |
| Folate, DFE (µg) | 543 | 8 | 546 | 12 | 494 | 12 | <0.0001 |
| Vitamin B6 (mg) | 2.23 | 0.04 | 2.29 | 0.05 | 2.06 | 0.05 | 0.0024 |
| Vitamin B12 (µg) | 4.80 | 0.12 | 5.15 | 0.14 | 5.10 | 0.14 | 0.9071 |
| Vitamin C (mg) | 85.6 | 1.7 | 82.8 | 2.5 | 66.2 | 2.3 | <0.0001 |
| Vitamin D (µg) | 4.52 | 0.10 | 4.82 | 0.18 | 4.11 | 0.12 | <0.0001 |
| Vitamin E, ATE (mg) | 9.76 | 0.18 | 9.48 | 0.24 | 8.26 | 0.19 | <0.0001 |
| Dietary fiber (g) | 19.3 | 0.3 | 17.8 | 0.3 | 14.7 | 0.3 | <0.0001 |
| Potassium (mg) | 2833 | 30 | 2762 | 41 | 2446 | 36 | <0.0001 |
| Sodium (mg) | 3637 | 40 | 3630 | 45 | 3393 | 57 | <0.0001 |
| Choline (mg) | 362 | 5 | 341 | 5 | 302 | 5 | <0.0001 |
| Adults (aged 60+ years) | | | | | | | |
| Calcium (mg) | 893 | 16 | 905 | 19 | 826 | 20 | 0.0053 |
| Copper (mg) | 1.32 | 0.04 | 1.29 | 0.04 | 1.07 | 0.02 | 0.0000 |
| Iron (mg) | 14.2 | 0.2 | 14.7 | 0.3 | 13.5 | 0.3 | 0.0507 |
| Magnesium (mg) | 307 | 4 | 295 | 5 | 253 | 4 | <0.0001 |
| Phosphorus (mg) | 1321 | 17 | 1313 | 21 | 1161 | 20 | <0.0001 |
| Selenium (µg) | 110 | 1 | 107 | 2 | 92.4 | 1.7 | <0.0001 |
| Zinc (mg) | 10.8 | 0.2 | 10.9 | 0.2 | 9.38 | 0.19 | <0.0001 |
| Vitamin A, RE (µg) | 725 | 35 | 714 | 26 | 609 | 18 | 0.0043 |
| Thiamin (mg) | 1.54 | 0.02 | 1.57 | 0.03 | 1.41 | 0.03 | 0.0561 |
| Riboflavin (mg) | 2.08 | 0.03 | 2.13 | 0.04 | 1.90 | 0.04 | 0.0077 |
| Niacin (mg) | 23.7 | 0.3 | 23.3 | 0.4 | 21.6 | 0.7 | 0.2162 |
| Folate, DFE (µg) | 497 | 9 | 504 | 10 | 454 | 14 | 0.0012 |
| Vitamin B6 (mg) | 2.04 | 0.04 | 1.93 | 0.04 | 1.87 | 0.11 | 0.5760 |
| Vitamin B12 (µg) | 4.94 | 0.24 | 5.09 | 0.22 | 4.51 | 0.20 | 0.1678 |
| Vitamin C (mg) | 88.5 | 2.4 | 81.9 | 2.9 | 70.6 | 2.3 | <0.0001 |
| Vitamin D (µg) | 5.00 | 0.14 | 4.89 | 0.17 | 4.40 | 0.20 | 0.0098 |
| Vitamin E, ATE (mg) | 8.88 | 0.18 | 9.23 | 0.25 | 7.64 | 0.20 | <0.0001 |
| Dietary fiber (g) | 18.4 | 0.3 | 17.2 | 0.4 | 14.6 | 0.3 | <0.0001 |
| Potassium (mg) | 2772 | 38 | 2685 | 41 | 2365 | 35 | <0.0001 |
| Sodium (mg) | 3202 | 42 | 3224 | 49 | 2893 | 57 | 0.0012 |
| Choline (mg) | 333 | 4 | 324 | 5 | 283 | 5 | <0.0001 |

Regression analysis was conducted with added sugars as percentage of calories as a continuous variable determined as the average of two days of intake adjusting for the complex sample design of NHANES and using 2-day dietary weights. ATE: alpha tocopherol equivalents; DFE: dietary folate equivalents; RE: retinol activity equivalents.

Supplemental Table 4. Percentage of the population below the EAR/above the AI for those with <10, 10-15, and >15% of calories as added sugars (AS10, AS10-15 and AS15) in children and adults by age groups - NHANES 2011-2018 data, gender combined population.

|  | Calories from Added Sugars (Added Sugars Intake Categories) | | | | | | | P values for comparison | | |
| --- | --- | --- | --- | --- | --- | --- | --- | --- | --- | --- |
|  | <10%  (AS10) | | 10% to 15%  (AS10-15) | | | >15%  (AS15) | | <10% vs 10-15%  (AS10 vs AS10-15) | <10% vs >15%  (AS10 vs AS15) | 10-15% vs >15%  (AS10-15 vs AS15) |
|  | Mean | SE | Mean | SE | Mean | | SE |  |  |  |
| Children (aged 2-4 years) | | | | | | | | | | |
| % below Estimated Average Requirement (EAR) | | | | | | | |  |  |  |
| Calcium | 10.68 | 1.35 | 14.26 | 1.82 | 19.88 | | 2.71 | 0.1136 | 0.0024 | 0.0854 |
| Copper | 0.08 | 0.05 | 0.07 | 0.06 | 0.09 | | 0.11 | 0.9358 | 0.9336 | 0.8953 |
| Iron | 1.27 | 0.29 | 0.98 | 0.24 | 1.90 | | 0.52 | 0.4503 | 0.2819 | 0.1048 |
| Magnesium | 0.49 | 0.24 | 0.52 | 0.24 | 0.68 | | 0.58 | 0.9253 | 0.7632 | 0.8009 |
| Phosphorus | 0.18 | 0.11 | 0.03 | 0.03 | 0.20 | | 0.14 | 0.2147 | 0.9077 | 0.2395 |
| Selenium | 0.00 | 0.01 | 0.00 | 0.01 | 0.00 | | 0.01 | 1.0000 | 1.0000 | 1.0000 |
| Zinc | 0.67 | 0.31 | 0.26 | 0.17 | 1.30 | | 0.71 | 0.2558 | 0.4207 | 0.1585 |
| Vitamin A, RE | 2.92 | 0.84 | 0.72 | 0.35 | 4.18 | | 1.21 | 0.0156 | 0.3903 | 0.0060 |
| Thiamin | 0.04 | 0.05 | 0.02 | 0.04 | 0.47 | | 0.25 | 0.6572 | 0.0938 | 0.0739 |
| Riboflavin | 0.05 | 0.03 | 0.00 | 0.01 | 0.10 | | 0.09 | 0.1290 | 0.6491 | 0.2936 |
| Niacin | 0.05 | 0.04 | 0.01 | 0.02 | 0.48 | | 0.30 | 0.3962 | 0.1537 | 0.1196 |
| Folate, DFE | 0.09 | 0.10 | 0.07 | 0.09 | 0.41 | | 0.33 | 0.8957 | 0.3493 | 0.3170 |
| Vitamin B6 | 0.04 | 0.05 | 0.00 | 0.01 | 0.55 | | 0.48 | 0.4030 | 0.2963 | 0.2547 |
| Vitamin B12 | 0.19 | 0.13 | 0.02 | 0.05 | 0.36 | | 0.22 | 0.2262 | 0.5011 | 0.1344 |
| Vitamin C | 0.27 | 0.18 | 0.69 | 0.38 | 0.38 | | 0.27 | 0.3202 | 0.7559 | 0.5013 |
| Vitamin D | 83.32 | 1.78 | 92.20 | 1.61 | 93.92 | | 1.98 | 0.0002 | 0.0001 | 0.4983 |
| Vitamin E, ATE | 43.12 | 3.03 | 38.81 | 3.91 | 46.51 | | 4.08 | 0.3844 | 0.5045 | 0.1733 |
| % above Adequate Intake (AI) | | | | | | | |  |  |  |
| Dietary fiber | 6.12 | 1.31 | 2.04 | 0.66 | 0.37 | | 0.28 | 0.0054 | <0.0001 | 0.0204 |
| Potassium | 49.42 | 2.53 | 36.13 | 2.89 | 27.56 | | 2.24 | 0.0005 | <0.0001 | 0.0189 |
| Sodium | 99.89 | 0.06 | 99.94 | 0.08 | 99.70 | | 0.16 | 0.6421 | 0.2573 | 0.1735 |
| Choline | 59.07 | 2.94 | 46.68 | 3.84 | 35.63 | | 5.17 | 0.0105 | 0.0001 | 0.0861 |
| Children (aged 5-8 years) | | | | | | | | | | |
| % below Estimated Average Requirement (EAR) | | | | | | | |  |  |  |
| Calcium | 20.03 | 1.64 | 18.40 | 2.61 | 33.10 | | 2.74 | 0.5974 | <0.0001 | 0.0001 |
| Copper | 0.02 | 0.03 | 0.02 | 0.03 | 0.09 | | 0.05 | 0.9491 | 0.2557 | 0.2049 |
| Iron | 0.87 | 0.25 | 0.79 | 0.18 | 1.43 | | 0.35 | 0.7962 | 0.1874 | 0.0992 |
| Magnesium | 0.56 | 0.29 | 0.22 | 0.13 | 0.74 | | 0.39 | 0.2886 | 0.7102 | 0.2046 |
| Phosphorus | 0.05 | 0.04 | 0.01 | 0.01 | 0.04 | | 0.03 | 0.2585 | 0.8671 | 0.2889 |
| Selenium | 0.00 | 0.00 | 0.00 | 0.00 | 0.00 | | 0.00 | 1.0000 | 1.0000 | 1.0000 |
| Zinc | 0.56 | 0.27 | 0.12 | 0.09 | 1.43 | | 0.59 | 0.1265 | 0.1833 | 0.0292 |
| Vitamin A, RE | 3.63 | 0.89 | 0.73 | 0.43 | 3.38 | | 1.19 | 0.0033 | 0.8669 | 0.0370 |
| Thiamin | 0.02 | 0.02 | 0.00 | 0.01 | 0.11 | | 0.08 | 0.4992 | 0.2885 | 0.1980 |
| Riboflavin | 0.03 | 0.03 | 0.00 | 0.00 | 0.03 | | 0.04 | 0.2978 | 0.9559 | 0.4259 |
| Niacin | 0.02 | 0.02 | 0.00 | 0.00 | 0.12 | | 0.09 | 0.3628 | 0.2927 | 0.2066 |
| Folate, DFE | 0.02 | 0.04 | 0.03 | 0.03 | 0.13 | | 0.13 | 0.8315 | 0.4367 | 0.4812 |
| Vitamin B6 | 0.01 | 0.06 | 0.00 | 0.01 | 0.44 | | 0.28 | 0.8181 | 0.1328 | 0.1132 |
| Vitamin B12 | 0.25 | 0.14 | 0.03 | 0.03 | 0.24 | | 0.16 | 0.1396 | 0.9428 | 0.2228 |
| Vitamin C | 1.13 | 0.54 | 1.18 | 0.53 | 1.63 | | 0.84 | 0.9468 | 0.6175 | 0.6523 |
| Vitamin D | 88.61 | 2.14 | 91.94 | 1.71 | 93.98 | | 1.23 | 0.2235 | 0.0297 | 0.3327 |
| Vitamin E, ATE | 33.69 | 3.02 | 25.67 | 3.64 | 36.85 | | 3.46 | 0.0900 | 0.4898 | 0.0259 |
| % above Adequate Intake (AI) | | | | | | | |  |  |  |
| Dietary fiber | 5.20 | 1.33 | 1.43 | 0.55 | 0.10 | | 0.06 | 0.0088 | 0.0001 | 0.0157 |
| Potassium | 46.85 | 2.76 | 36.52 | 2.76 | 24.11 | | 2.71 | 0.0080 | <0.0001 | 0.0013 |
| Sodium | 99.98 | 0.02 | 100.00 | 0.01 | 99.97 | | 0.03 | 0.5018 | 0.8605 | 0.5466 |
| Choline | 53.21 | 2.51 | 44.44 | 3.69 | 26.04 | | 3.02 | 0.0499 | <0.0001 | 0.0001 |
| Children (aged 9-13 years) | | | | | | | | | | |
| % below Estimated Average Requirement (EAR) | | | | | | | |  |  |  |
| Calcium | 54.83 | 3.08 | 56.21 | 2.93 | 73.18 | | 2.37 | 0.7451 | <0.0001 | <0.0001 |
| Copper | 3.13 | 1.19 | 1.39 | 0.39 | 3.06 | | 0.93 | 0.1646 | 0.9635 | 0.0980 |
| Iron | 0.58 | 0.31 | 0.14 | 0.10 | 0.60 | | 0.26 | 0.1876 | 0.9479 | 0.0957 |
| Magnesium | 23.30 | 3.48 | 18.94 | 2.06 | 34.83 | | 3.44 | 0.2810 | 0.0183 | 0.0001 |
| Phosphorus | 21.37 | 3.15 | 16.25 | 1.99 | 33.80 | | 2.57 | 0.1691 | 0.0022 | <0.0001 |
| Selenium | 0.01 | 0.03 | 0.00 | 0.01 | 0.00 | | 0.01 | 0.7382 | 0.7722 | 0.8926 |
| Zinc | 13.58 | 2.92 | 6.08 | 1.91 | 17.82 | | 2.79 | 0.0318 | 0.2937 | 0.0005 |
| Vitamin A, RE | 23.71 | 3.37 | 10.20 | 2.56 | 28.05 | | 2.87 | 0.0014 | 0.3277 | <0.0001 |
| Thiamin | 0.36 | 0.20 | 0.09 | 0.08 | 0.78 | | 0.41 | 0.2071 | 0.3663 | 0.1021 |
| Riboflavin | 1.00 | 0.51 | 0.06 | 0.08 | 1.01 | | 0.48 | 0.0668 | 0.9895 | 0.0506 |
| Niacin | 0.21 | 0.13 | 0.03 | 0.03 | 0.75 | | 0.38 | 0.1498 | 0.1818 | 0.0583 |
| Folate, DFE | 1.66 | 0.75 | 0.86 | 0.39 | 2.00 | | 0.77 | 0.3401 | 0.7556 | 0.1865 |
| Vitamin B6 | 1.43 | 0.67 | 0.16 | 0.15 | 4.27 | | 1.31 | 0.0636 | 0.0530 | 0.0018 |
| Vitamin B12 | 1.56 | 0.67 | 0.27 | 0.22 | 1.63 | | 0.74 | 0.0677 | 0.9418 | 0.0778 |
| Vitamin C | 15.24 | 3.48 | 11.31 | 2.26 | 19.38 | | 3.23 | 0.3440 | 0.3834 | 0.0406 |
| Vitamin D | 90.98 | 1.68 | 95.18 | 1.15 | 97.33 | | 0.79 | 0.0397 | 0.0006 | 0.1242 |
| Vitamin E, ATE | 74.06 | 4.63 | 66.99 | 3.84 | 78.91 | | 3.34 | 0.2405 | 0.3954 | 0.0193 |
| % above Adequate Intake (AI) | | | | | | | |  |  |  |
| Dietary fiber | 1.97 | 0.72 | 0.67 | 0.34 | 0.02 | | 0.03 | 0.1055 | 0.0070 | 0.0565 |
| Potassium | 41.42 | 3.59 | 39.14 | 3.03 | 20.05 | | 2.18 | 0.6275 | <0.0001 | <0.0001 |
| Sodium | 99.97 | 0.03 | 99.98 | 0.02 | 99.94 | | 0.05 | 0.8452 | 0.5400 | 0.4195 |
| Choline | 13.03 | 2.42 | 7.95 | 2.01 | 2.17 | | 0.91 | 0.1064 | <0.0001 | 0.0087 |
| Children (aged 14-18 years) | | | | | | | | | | |
| % below Estimated Average Requirement (EAR) | | | | | | | |  |  |  |
| Calcium | 54.41 | 2.59 | 67.03 | 2.36 | 80.18 | | 2.68 | 0.0003 | <0.0001 | 0.0002 |
| Copper | 7.55 | 1.52 | 9.13 | 1.82 | 17.98 | | 2.77 | 0.5041 | 0.0010 | 0.0076 |
| Iron | 5.16 | 0.81 | 5.85 | 1.05 | 9.70 | | 1.67 | 0.6010 | 0.0146 | 0.0516 |
| Magnesium | 75.27 | 4.76 | 89.23 | 2.21 | 96.74 | | 1.18 | 0.0079 | <0.0001 | 0.0027 |
| Phosphorus | 16.88 | 1.69 | 19.24 | 2.61 | 37.27 | | 2.92 | 0.4476 | <0.0001 | <0.0001 |
| Selenium | 0.07 | 0.07 | 0.05 | 0.06 | 0.07 | | 0.09 | 0.8757 | 0.9345 | 0.8282 |
| Zinc | 15.19 | 2.64 | 17.85 | 3.57 | 33.08 | | 4.10 | 0.5493 | 0.0002 | 0.0051 |
| Vitamin A, RE | 45.71 | 2.90 | 53.55 | 3.94 | 68.72 | | 2.63 | 0.1090 | <0.0001 | 0.0014 |
| Thiamin | 2.33 | 0.70 | 2.09 | 0.93 | 8.04 | | 2.06 | 0.8407 | 0.0087 | 0.0086 |
| Riboflavin | 2.63 | 0.65 | 1.37 | 0.70 | 4.82 | | 1.25 | 0.1853 | 0.1209 | 0.0161 |
| Niacin | 0.41 | 0.20 | 0.15 | 0.15 | 1.61 | | 0.69 | 0.3111 | 0.0934 | 0.0389 |
| Folate, DFE | 6.28 | 1.43 | 8.55 | 2.27 | 19.15 | | 3.62 | 0.3959 | 0.0009 | 0.0131 |
| Vitamin B6 | 3.68 | 1.07 | 2.19 | 1.02 | 10.78 | | 2.30 | 0.3135 | 0.0051 | 0.0006 |
| Vitamin B12 | 4.42 | 1.03 | 2.99 | 1.21 | 4.04 | | 1.11 | 0.3685 | 0.8029 | 0.5224 |
| Vitamin C | 42.14 | 3.92 | 50.94 | 4.74 | 58.48 | | 4.45 | 0.1525 | 0.0059 | 0.2469 |
| Vitamin D | 93.36 | 1.43 | 98.67 | 0.48 | 98.79 | | 0.44 | 0.0004 | 0.0003 | 0.8552 |
| Vitamin E, ATE | 89.65 | 4.09 | 95.87 | 1.51 | 97.53 | | 0.93 | 0.1535 | 0.0600 | 0.3465 |
| % above Adequate Intake (AI) | | | | | | | |  |  |  |
| Dietary fiber | 1.49 | 0.72 | 0.30 | 0.19 | 0.00 | | 0.01 | 0.1102 | 0.0387 | 0.1064 |
| Potassium | 33.38 | 3.11 | 19.33 | 2.35 | 10.50 | | 1.72 | 0.0003 | 0.0000 | 0.0024 |
| Sodium | 99.88 | 0.08 | 99.88 | 0.08 | 99.59 | | 0.20 | 0.9862 | 0.1764 | 0.1746 |
| Choline | 5.50 | 2.02 | 0.62 | 0.34 | 0.15 | | 0.09 | 0.0173 | 0.0081 | 0.1709 |
| Adults (aged 19-30 years) | | | | | | | | | | |
| % below Estimated Average Requirement (EAR) | | | | | | | |  |  |  |
| Calcium | 26.10 | 2.00 | 23.43 | 2.57 | 43.73 | | 2.22 | 0.4119 | <0.0001 | <0.0001 |
| Copper | 5.58 | 0.76 | 4.71 | 1.13 | 18.69 | | 2.05 | 0.5243 | <0.0001 | <0.0001 |
| Iron | 8.24 | 0.65 | 6.99 | 0.83 | 11.40 | | 0.96 | 0.2340 | 0.0064 | 0.0005 |
| Magnesium | 39.13 | 2.36 | 50.20 | 3.21 | 74.19 | | 2.07 | 0.0055 | <0.0001 | <0.0001 |
| Phosphorus | 0.41 | 0.11 | 0.12 | 0.06 | 1.41 | | 0.49 | 0.0254 | 0.0469 | 0.0091 |
| Selenium | 0.14 | 0.06 | 0.04 | 0.05 | 1.00 | | 0.38 | 0.1939 | 0.0259 | 0.0121 |
| Zinc | 13.17 | 1.39 | 10.20 | 1.96 | 24.49 | | 2.02 | 0.2173 | <0.0001 | <0.0001 |
| Vitamin A, RE | 41.45 | 2.56 | 48.34 | 3.14 | 66.44 | | 2.23 | 0.0889 | <0.0001 | <0.0001 |
| Thiamin | 4.57 | 0.77 | 3.29 | 0.83 | 9.77 | | 1.42 | 0.2569 | 0.0013 | 0.0001 |
| Riboflavin | 2.69 | 0.48 | 1.92 | 0.57 | 8.19 | | 1.21 | 0.2990 | <0.0001 | <0.0001 |
| Niacin | 0.44 | 0.16 | 0.24 | 0.14 | 1.96 | | 0.61 | 0.3441 | 0.0161 | 0.0059 |
| Folate, DFE | 7.91 | 1.23 | 7.39 | 1.39 | 19.03 | | 1.59 | 0.7802 | <0.0001 | <0.0001 |
| Vitamin B6 | 2.72 | 0.64 | 3.79 | 1.08 | 10.50 | | 1.53 | 0.3897 | <0.0001 | 0.0003 |
| Vitamin B12 | 4.23 | 0.86 | 2.70 | 0.80 | 6.05 | | 1.02 | 0.1921 | 0.1724 | 0.0098 |
| Vitamin C | 45.14 | 2.30 | 52.15 | 3.01 | 65.96 | | 2.06 | 0.0643 | <0.0001 | 0.0002 |
| Vitamin D | 94.34 | 1.21 | 96.47 | 1.02 | 98.41 | | 0.47 | 0.1782 | 0.0017 | 0.0836 |
| Vitamin E, ATE | 74.45 | 2.24 | 78.61 | 2.86 | 89.96 | | 1.31 | 0.2528 | <0.0001 | 0.0003 |
| % above Adequate Intake (AI) | | | | | | | |  |  |  |
| Dietary fiber | 6.40 | 0.92 | 1.79 | 0.57 | 0.27 | | 0.11 | 0.0000 | <0.0001 | 0.0086 |
| Potassium | 32.92 | 2.32 | 22.73 | 2.34 | 11.06 | | 1.42 | 0.0020 | <0.0001 | <0.0001 |
| Sodium | 99.65 | 0.14 | 99.94 | 0.05 | 99.36 | | 0.23 | 0.0476 | 0.2838 | 0.0127 |
| Choline | 13.74 | 1.72 | 2.62 | 0.85 | 1.79 | | 0.49 | <0.0001 | <0.0001 | 0.3958 |
| Adults (aged 31-59 years) | | | | | | | | | | |
| % below Estimated Average Requirement (EAR) | | | | | | | |  |  |  |
| Calcium | 36.92 | 1.27 | 31.80 | 2.27 | 45.40 | | 1.94 | 0.0494 | 0.0003 | <0.0001 |
| Copper | 4.32 | 0.55 | 3.25 | 0.62 | 11.17 | | 1.19 | 0.1989 | <0.0001 | <0.0001 |
| Iron | 5.01 | 0.38 | 4.88 | 0.60 | 8.46 | | 0.83 | 0.8577 | 0.0002 | 0.0005 |
| Magnesium | 38.98 | 1.55 | 47.63 | 1.96 | 67.80 | | 1.91 | 0.0005 | <0.0001 | <0.0001 |
| Phosphorus | 0.41 | 0.10 | 0.11 | 0.07 | 1.14 | | 0.34 | 0.0159 | 0.0400 | 0.0033 |
| Selenium | 0.21 | 0.09 | 0.08 | 0.07 | 1.00 | | 0.36 | 0.2380 | 0.0345 | 0.0121 |
| Zinc | 12.99 | 1.11 | 11.80 | 1.85 | 20.73 | | 1.69 | 0.5803 | 0.0001 | 0.0004 |
| Vitamin A, RE | 39.49 | 1.84 | 41.91 | 2.62 | 54.47 | | 2.25 | 0.4486 | 0.0000 | 0.0003 |
| Thiamin | 5.47 | 0.65 | 3.70 | 0.86 | 9.12 | | 1.32 | 0.1001 | 0.0132 | 0.0006 |
| Riboflavin | 2.18 | 0.32 | 1.14 | 0.28 | 4.35 | | 0.64 | 0.0148 | 0.0024 | 0.0000 |
| Niacin | 0.56 | 0.15 | 0.43 | 0.16 | 2.51 | | 0.62 | 0.5604 | 0.0023 | 0.0012 |
| Folate, DFE | 9.43 | 1.04 | 9.75 | 1.57 | 18.42 | | 1.98 | 0.8644 | 0.0001 | 0.0006 |
| Vitamin B6 | 6.09 | 0.73 | 6.50 | 1.39 | 15.50 | | 1.71 | 0.7979 | <0.0001 | <0.0001 |
| Vitamin B12 | 4.93 | 0.78 | 4.15 | 1.04 | 6.70 | | 0.95 | 0.5501 | 0.1483 | 0.0697 |
| Vitamin C | 41.82 | 1.71 | 45.43 | 2.01 | 60.40 | | 2.27 | 0.1714 | <0.0001 | <0.0001 |
| Vitamin D | 95.85 | 0.64 | 94.99 | 0.95 | 97.09 | | 0.54 | 0.4548 | 0.1364 | 0.0553 |
| Vitamin E, ATE | 72.36 | 1.50 | 79.23 | 2.18 | 86.17 | | 1.71 | 0.0096 | <0.0001 | 0.0123 |
| % above Adequate Intake (AI) | | | | | | | |  |  |  |
| Dietary fiber | 12.90 | 0.97 | 5.94 | 1.12 | 1.31 | | 0.41 | <0.0001 | <0.0001 | 0.0001 |
| Potassium | 41.19 | 1.38 | 34.20 | 1.94 | 22.07 | | 1.63 | 0.0033 | <0.0001 | <0.0001 |
| Sodium | 99.55 | 0.13 | 99.88 | 0.07 | 98.86 | | 0.30 | 0.0254 | 0.0359 | 0.0011 |
| Choline | 14.22 | 1.16 | 5.14 | 1.24 | 3.86 | | 0.88 | <0.0001 | <0.0001 | 0.4019 |
| Adults (aged 60+ years) | | | | | | | | | | |
| % below Estimated Average Requirement (EAR) | | | | | | | |  |  |  |
| Calcium | 59.26 | 1.62 | 57.52 | 2.64 | 64.96 | | 2.52 | 0.5739 | 0.0571 | 0.0414 |
| Copper | 6.35 | 0.71 | 3.36 | 0.74 | 11.62 | | 1.42 | 0.0035 | 0.0009 | <0.0001 |
| Iron | 0.54 | 0.17 | 0.41 | 0.17 | 1.49 | | 0.56 | 0.6000 | 0.1012 | 0.0626 |
| Magnesium | 49.96 | 1.45 | 55.33 | 2.58 | 72.56 | | 1.65 | 0.0696 | <0.0001 | <0.0001 |
| Phosphorus | 0.93 | 0.20 | 0.26 | 0.13 | 2.28 | | 0.66 | 0.0064 | 0.0495 | 0.0027 |
| Selenium | 0.56 | 0.17 | 0.25 | 0.15 | 1.95 | | 0.60 | 0.1695 | 0.0254 | 0.0057 |
| Zinc | 17.91 | 1.49 | 15.90 | 2.03 | 31.04 | | 2.65 | 0.4234 | <0.0001 | <0.0001 |
| Vitamin A, RE | 33.71 | 1.74 | 34.14 | 3.22 | 47.21 | | 2.91 | 0.9067 | 0.0001 | 0.0026 |
| Thiamin | 7.27 | 0.86 | 5.50 | 1.08 | 13.16 | | 1.72 | 0.1980 | 0.0022 | 0.0002 |
| Riboflavin | 2.73 | 0.41 | 1.51 | 0.35 | 6.09 | | 1.10 | 0.0233 | 0.0043 | 0.0001 |
| Niacin | 1.41 | 0.31 | 1.42 | 0.44 | 5.80 | | 1.23 | 0.9920 | 0.0005 | 0.0008 |
| Folate, DFE | 13.03 | 1.21 | 13.00 | 1.64 | 22.84 | | 2.46 | 0.9881 | 0.0003 | 0.0009 |
| Vitamin B6 | 13.38 | 1.22 | 18.44 | 2.49 | 26.99 | | 2.49 | 0.0680 | <0.0001 | 0.0152 |
| Vitamin B12 | 4.83 | 0.95 | 4.36 | 1.14 | 8.23 | | 1.46 | 0.7493 | 0.0512 | 0.0369 |
| Vitamin C | 37.39 | 1.91 | 41.15 | 2.78 | 50.35 | | 2.27 | 0.2644 | <0.0001 | 0.0104 |
| Vitamin D | 92.64 | 0.92 | 94.27 | 0.76 | 95.03 | | 0.96 | 0.1726 | 0.0721 | 0.5349 |
| Vitamin E, ATE | 78.28 | 1.27 | 80.54 | 2.11 | 89.29 | | 1.52 | 0.3595 | <0.0001 | <0.0001 |
| % above Adequate Intake (AI) | | | | | | | |  |  |  |
| Dietary fiber | 19.86 | 1.29 | 11.55 | 1.65 | 3.95 | | 0.67 | 0.0001 | <0.0001 | <0.0001 |
| Potassium | 39.94 | 1.58 | 32.54 | 2.46 | 20.21 | | 1.74 | 0.0115 | <0.0001 | <0.0001 |
| Sodium | 98.93 | 0.26 | 99.49 | 0.20 | 97.12 | | 0.80 | 0.0855 | 0.0318 | 0.0042 |
| Choline | 10.45 | 0.83 | 3.88 | 1.06 | 2.76 | | 0.56 | <0.0001 | <0.0001 | 0.3526 |

Regression analysis was conducted assessing added sugars as categorical variables (AS10, AS10-15 and AS>15) determined as the average of two days of intake adjusting for the complex sample design of NHANES and using 2-day dietary weights with Z-tests to evaluate differences in groups. ATE: alpha tocopherol equivalents; DFE: dietary folate equivalents; RE: retinol activity equivalents.
